# Identification of Novel Type III Secretion Chaperone-Substrate Complexes of *Chlamydia trachomatis*

**DOI:** 10.1371/journal.pone.0056292

**Published:** 2013-02-19

**Authors:** Sara V. Pais, Catarina Milho, Filipe Almeida, Luís Jaime Mota

**Affiliations:** 1 Infection Biology Laboratory, Instituto de Tecnologia Química e Biológica, Universidade Nova de Lisboa, Oeiras, Portugal; 2 Departamento de Ciências da Vida, Faculdade de Ciências e Tecnologia, Universidade Nova de Lisboa, Caparica, Portugal; University of California Merced, United States of America

## Abstract

*Chlamydia trachomatis* is an obligate intracellular bacterial pathogen of humans that uses a type III secretion (T3S) system to manipulate host cells through the delivery of effector proteins into their cytosol and membranes. The function of T3S systems depends on small bacterial cytosolic chaperone-like proteins, which bind T3S substrates and ensure their appropriate secretion. To find novel T3S chaperone-substrate complexes of *C. trachomatis* we first searched its genome for genes encoding proteins with features of T3S chaperones. We then systematically tested for interactions between candidate chaperones and chlamydial T3S substrates by bacterial two-hybrid. This revealed interactions between Slc1 (a known T3S chaperone) or CT584 and several T3S substrates. Co-immunoprecipation after protein expression in *Yersinia enterocolitica* and protein overlay binding assays indicated that Slc1 interacted with the N-terminal region of the known T3S substrates Tarp (a previously described substrate of Slc1), CT694, and CT695, and that CT584 interacted with a central region of CT082, which we identified as a *C. trachomatis* T3S substrate using *Y. enterocolitica* as a heterologous system. Further T3S assays in *Yersinia* indicated that Slc1 or CT584 increased the amount of secreted Tarp, CT694, and CT695, or CT082, respectively. Expression of CT584 increased the intra-bacterial stability of CT082, while Slc1 did not affect the stability of its substrates. Overall, this indicated that in *C. trachomatis* Slc1 is a chaperone of multiple T3S substrates and that CT584 is a chaperone of the newly identified T3S substrate CT082.

## Introduction

Type III secretion (T3S) systems are used by many Gram-negative bacterial pathogens to manipulate eukaryotic host cells through the delivery of effector proteins into their cytosol and membranes [1]. The T3S machine (an injectisome) is a multi-protein complex which is evolutionarily related to the bacterial flagellum. It consists in a basal body embedded within bacterial membranes usually topped by a needle-like structure, which in some cases is extended by a filament. T3S substrates include: (i) effectors; (ii) translocators, comprising two proteins assembling a pore in a host cell membrane (that mediates translocation of effectors), and one protein forming a needle tip complex or a filament (linking the needle to the translocation pore); (iii) injectisome components; (iv) regulatory proteins.

Secretion of some, but not all, T3S substrates requires the assistance in the bacterial cytosol of characteristic chaperone-like proteins [1]–[3]. T3S chaperones are generally small (15–20 kDa), acidic [isoelectric point (pI)<6], and dimeric proteins that remain in the cytosol after substrate secretion. They can assist secretion of their substrates by several mechanisms [1], [3]–[9]. T3S chaperones have been divided into those that bind effectors (class I), pore forming translocators (class II), or subunits of injectisome or flagellar substructures (class III) [1], [10]. Class I chaperones are the best studied. They share low sequence similarity between each other but display a conserved three-dimensional (3D) structure.

*Chlamydia trachomatis* causes ocular and genital infections in humans that are a significant clinical and public health concern [11], [12]. *C. trachomatis* is part of a family of obligate intracellular bacteria sharing a characteristic developmental cycle that involves the inter-conversion between an infectious form, the elementary body (EB), and a non-infectious form, the reticulate body (RB) [13]. Throughout development, *Chlamydiae* reside and multiply within a membrane-bound compartment, the inclusion, and use a T3S system to translocate effector proteins into host cells [14], [15]. In spite of recent breakthroughs [16]–[19], studies on molecular aspects of *C. trachomatis* infections are still hampered by the lack of straightforward methods for its genetic manipulation.

Numerous *C. trachomatis* T3S substrates have been found by using *Salmonella* [20], *Shigella* [21], [22], or *Yersinia* [23]–[25] as heterologous hosts, or suggested by machine learning algorithms based on the N-terminal T3S signal of known effectors [26]–[28]. Based on this, it has been estimated that *C. trachomatis* genomes may encode ∼100 T3S substrates [29], [30]. *C. trachomatis* T3S effectors have been identified by immunofluorescence microscopy using specific antibodies against chlamydial proteins, which enabled to visualize their translocation during host cell infection. These include Tarp [24], CT694 [25], CopN [23], Cap1 [31], CT620 and CT621 [22], [32], and several proteins containing a hydrophobic motif thought to mediate their insertion into the inclusion membrane (Inc proteins) [33], [34]. However, only two *C. trachomatis* class I chaperones have been identified: CT260 (known as Mcsc), which binds to and stabilizes at least Cap1 and the Inc protein CT618 [29], and CT043 (known as Slc1), the chaperone of Tarp [35]. In addition, CP0432 (Scc1) and CP0033 (Scc4) (the *Chlamydia pneumoniae* orthologs of *C. trachomatis* CT088 and CT663, respectively) function as a heterodimeric chaperone of CopN [36]. *C. trachomatis* class II and class III chaperones have also been identified [37], [38].

In this work, we found that Slc1 chaperones not only Tarp [35], but also CT694 and the T3S substrate CT695 [25], and that CT584 binds to, stabilizes, and promotes T3S of CT082, a newly identified *C. trachomatis* type III substrate.

## Materials and Methods

### Cell Culture, Bacterial Strains and Growth Conditions

HeLa 229 (ATCC) and HeLa HtTA1 (European Collection of Cell Cultures; ECACC) cells were maintained in Dulbecco’s modified Eagle’s medium (DMEM; Invitrogen) supplemented with 10% (v/v) foetal bovine serum (Invitrogen) at 37°C in a humidified atmosphere of 5% (v/v) CO_2_. HeLa HtTA1 cells were transfected with plasmid DNA by using the jetPEI reagent (Polyplus Transfection), as detailed by the manufacturer.

*C. trachomatis* L2/434 (from ATCC) was propagated in HeLa 229 cells using standard techniques [39]. *Escherichia coli* TOP10 (Invitrogen) was used for construction and purification of the plasmids, *E. coli* BTH101 (Euromedex) was used for bacterial two-hybrid, and *E. coli* BL21 DE3 (Novagen) was used for protein expression. *Yersinia enterocolitica* ΔHOPEMT (MRS40 pIML421 [*yopH*_Δ1–352_, *yopO*_Δ65–558_, *yopP_23_*, *yopE_21_*, *yopM_23_*, *yopT_135_*]), deficient for the *Yersinia* Yop T3S effectors H, O, P, E, M, and T, but T3S-proficient [40] and T3S-deficient *Y. enterocolitica* ΔHOPEMT ΔYscU (MRS40 pFA1001 [*yopH*_Δ1–352_, *yopO*_Δ65–558_, *yopP_23_*, *yopE_21_*, *yopM_23_*, *yopT_135_*, *yscU_Δ1–354_*) [41] were used for T3S assays. The *yscU* gene encodes an essential component of the *Y. enterocolitica* T3S system, and the *yscU_Δ1–354_* mutation is non-polar [42]. *E. coli* or *Y. enterocolitica* were routinely grown in liquid or solid Luria-Bertani (LB) medium with the appropriate antibiotics and supplements. Plasmids were introduced into *E. coli* or *Y. enterocolitica* by electroporation.

### DNA Manipulations, Plasmids, and Primers

The plasmids used in this work and its main characteristics are detailed in Table S1. The DNA primers used are shown in Table S2. Plasmids were constructed using standard DNA manipulations with proof-reading Phusion DNA polymerase (Finnzymes), restriction enzymes (MBI Fermentas), T4 DNA Ligase (Invitrogen), DreamTaq DNA polymerase (MBI Fermentas), NucleoSpin Gel and PCR Clean-up kit (Macherey-Nagel), and purified with GeneElute Plasmid Miniprep kit (Sigma), according to the instructions of each manufacturers. *C. trachomatis* genes, or parts of genes, were amplified by PCR from genomic DNA of strain L2/434 using custom oligonucleotide primers (Table S2). The accuracy of the nucleotide sequence of all the inserts in the constructed plasmids was checked by DNA sequencing.

### Immunoblotting

The following antibodies were used for immunoblotting: rabbit polyclonal anti-Myc (ab9106; Abcam; 1∶1000), rat monoclonal anti-HA (3F10; Roche; 1∶1000), mouse monoclonal anti-TEM-1 (QED Bioscience; 1∶500), rabbit polyclonal anti-SycO (1∶1000) [4], mouse monoclonal anti-hexahistidine [(6x)His]-tag (HIS-1; Abcam; 1∶1500), rabbit polyclonal anti-GST (G7781; Sigma; 1∶5000), goat polyclonal anti-major outer membrane protein (MOMP) of *C. trachomatis* (ab34414; Abcam; 1∶1000), goat polyclonal anti-green fluorescent protein (GFP) (SICGEN; 1∶1000), and mouse monoclonal anti-α-tubulin (B-5-1-2; Sigma; 1∶1000). The generation and purification of the rabbit polyclonal anti-CT082 and anti-CT584 used is described below. Immunoblot detection was done with horseradish peroxidase-conjugated secondary antibodies (GE Healthcare and Jackson ImmunoResearch), Western Lightning *Plus*-ECL (Perkin Elmer), and a ChemiDoc XRS+ system (BioRad).

### Bacterial Two-hybrid

We used the bacterial adenylate cyclase two-hybrid (BACTH) system [43] (Euromedex). This system includes four plasmids (pUT18, pUT18C, pKNT25 and pKT25; Table S1), enabling fusions to the N- or C-terminus of T18 or T25 fragments of the catalytic domain of adenylate cyclase (CyaA) from *Bordetella pertussis*. For bacterial two-hybrid, adenylate cyclase (Cya)-deficient *E. coli* BTH101 was transformed with plasmids expressing the different T18 and T25 hybrid proteins. Co-transformants were grown on solid LB supplemented with isopropyl β-D-1-thiogalactopyranoside (IPTG; 0.5 mM), the chromogenic substrate 5-bromo-4-chloro-indolyl-β-D-galactoside (X-gal; 40 µg/ml) and antibiotics. Plates were incubated for 24 h at 28°C. The appearance of blue colonies, resulting from β-galactosidase (β-gal)-mediated cleavage of X-gal, was taken as a first indication of a protein-protein interaction. In these cases, we further quantified β-gal activity by monitoring the β-gal-dependent cleavage of *ortho*-nitrophenyl-β-galactoside (ONPG) into the colorimetric molecule *ortho*-nitrophenol, as described by the manufacturer (Euromedex). In all experiments, we used as positive control strain BTH101 transformed with plasmids pUT18C-zip and pKT25-zip, encoding two leucine zipper domains, and as negative control strain BTH101 transformed with plasmids pUT18C and pKT25. The final results were based on three independent experiments, using three different single bacterial colonies.

### Co-immunoprecipitation

*Y. enterocolitica* ΔHOPEMT strains carrying pBAD/*Myc*-His A (Invitrogen) derivatives encoding C-terminally Myc-tagged *C. trachomatis* T3S substrates (Tarp, CT082, CT621, CT694, and CT695) under the control of the *E. coli* arabinose operon promoter (*P_BAD_*), and pBBR1MCS-2 [44] derivatives encoding C-terminally hemagluttinin (HA)-tagged candidate T3S chaperones (Slc1 and CT584) under the control of the promoter of the gene encoding the *Y. enterocolitica* T3S chaperone SycE, were diluted from overnight cultures to an optical density at 600 nm (OD_600_) of 0.1 in brain heart infusion broth supplemented with 5 mM of CaCl_2_ and the appropriate antibiotics (non-secreting conditions). The bacterial cultures were incubated for 2 h at 26°C and at 150 rpm, and then shifted to 37°C (to induce expression from the *sycE* promoter) and incubated for more 4 h at 150 rpm. At the time of the temperature shift, to induce expression from *P_BAD_*, L-arabinose was added to the cultures to a final concentration of 0.2% (w/v). After the 4 h incubation at 37°C, bacterial cultures were transferred to ice and the equivalent of 5 OD_600_ units were centrifuged at 4,500 *g* for 10 min at 4°C. The bacterial cells were washed once with ice-cold phosphate buffered saline (PBS) and lysed in PBS using the BugBuster reagent (Novagen). After centrifugation at 17,000 *g* for 10 min at 4°C, the lysate supernatant was pre-cleared with 20 µl of Pierce Protein G agarose (Thermo Scientific), which had been previously washed with ice-cold co-immunoprecipitation (co-IP) buffer [20 mM Tris-HCl, pH 8; 150 mM NaCl; 1% (v/v) Triton X-100], by incubation for 1 h at 4°C with end-over-end rotation. A sample of the pre-cleared lysate was collected for further analysis (input), and the remaining was incubated with 10 µl of Pierce Protein G agarose (Thermo Scientific) that had been previously incubated with 5 µg of mouse monoclonal anti-Myc (9E10; Sigma) or with 5 µg of mouse monoclonal anti-HA (HA-7; Sigma) for 1 h 30 min at 4°C with end-over-end rotation. The IPs were carried out at 4°C with end-over-end rotation for 2 h. The samples were then centrifuged at 500 *g* for 2 min at 4°C and washed three-times with ice-cold co-IP buffer. Finally, 25 µl of Laemmli SDS-PAGE loading buffer were added to the agarose beads and the samples were incubated for 5 min at 95°C (output). Input and output samples were analysed by immunoblotting.

### *Y. enterocolitica* T3S Assays

T3S assays were done as previously described [42]. In these experiments, we used *Y. enterocolitica* ΔHOPEMT or ΔHOPEMT ΔYscU strains carrying the plasmids described above (in the co-immunoprecipitation section). In addition, we also used plasmids expressing C-terminally Myc-tagged *Yersinia* YopE and/or C-terminally HA-tagged *C. trachomatis* CT790. The amounts of protein in bacterial pellets and culture supernatants were analysed by immunoblotting and Coomassie staining. The amounts of protein in bacterial pellets and/or culture supernatants were estimated from images of immunoblots and of Coomassie-stained SDS-PAGE with Image Lab (Bio-Rad). Briefly, using “Volume tools” of Image Lab, the band to be quantified was manually selected and the background was subtracted using the local subtraction method. The quantification value was given by a mean value, which corresponds to all the pixels inside the volume boundary. These data are represented as the ratio between when T3S substrates (Tarp, CT082, CT621, CT694, and CT695 from *C. trachomatis*, and LcrV, YopB, YopD, YopE, YopN, and YscP from *Y. enterocolitica*) were expressed together with Slc1, CT584, or CT790, relative to when they were expressed alone. Because YopB, YopD, LcrV and YopN could not be easily distinguished as independent bands in Coomassie-stained SDS-PAGE, the amounts of these proteins in the supernatant fraction were quantified all together (as if they corresponded to a single secreted protein). The results from the quantifications are the average ± standard error of the mean (SEM) from three or four independent experiments, as indicated.

### Stability Assays

*Y. enterocolitica* ΔHOPEMT strains carrying plasmids encoding Myc-tagged *C. trachomatis* T3S substrates (Tarp, CT082, CT694, and CT695) and HA-tagged candidate T3S chaperones (Slc1 and CT584), were diluted from overnight cultures and incubated as described above for co-IP experiments. After 2 h (strains expressing CT694-Myc or CT695-Myc) or 4 h (strains expressing CT082-HA or Tarp-HA) of incubation at 37°C, chloramphenicol was added to a final concentration of 50 µg/ml. Samples were then taken at different time points and analysed by immunobloting.

### Protein Purification

*E. coli* BL21 strains harbouring plasmids encoding (6x)His-CT584 or a fusion of maltose binding protein to the N-terminus of CT082 (MBP-CT082) were grown at 37°C [(6x)His-CT584] or at 26°C [MBP-CT082] in auto-induction conditions [45]. The bacterial cells were harvested by centrifugation and ressuspended in 20 mM sodium phosphate buffer (pH 7.4), 500 mM NaCl, 10 mM imidazole [cells expressing (6x)His-CT584], or in 20 mM Tris-HCl (pH 7.4), 200 mM NaCl, 1 mM EDTA, and 1 mM DTT (cells expressing MBP-CT082). Cells were lyzed by passing twice through a French press in the presence of 1 mM phenylmethylsulphonyl fluoride (PMSF) and centrifuged at 15,000 *g* for 1 h at 4°C. His-tagged fusion proteins [(6x)His-CT584] or MBP-CT082 were purified from the soluble fraction using Ni-nitrilotriacetic acid (NTA) resin (Qiagen) or amylose resin (New England Biolabs), respectively, according to the instructions of the manufacturers.

#### Generation and purification of rabbit anti-CT082 and anti-CT584 antibodies

Purified (6x)His-CT584 and MBP-CT082 were used to immunize New Zealand White rabbits for the production of polyclonal antisera (Davids Biotechnologie, Regensburg, Germany). The anti-CT584 serum was affinity purified on (6x)His-CT584 immobilized on a nitrocellulose membrane. The anti-CT082 serum was first depleted of anti-MBP antibodies by repeated passages on an amylose resin with bound purified MBP, and then affinity purified on MBP-CT082 immobilized on a nitrocellulose membrane. The affinity purified antibodies were concentrated using Amicon Ultra-4 (Millipore) and quantified using NanoDrop 1000 (Thermo Scientific).

### Protein Overlay Binding Assays

*E. coli* BL21 DE3 strains harbouring plasmids encoding glutathione S-transferase (GST) or fusions of GST to the N-terminus of CT082 were grown at 37°C under auto-induction conditions [45]. Bacterial cells were collected and ressuspended in Laemmli SDS-PAGE loading buffer. Samples were resolved in two identical SDS-PAGE and then transferred onto nitrocellulose membranes. An overlay binding assay was then performed, as described [46]. Briefly, one of the membranes was incubated with 1.2 µg of (6x)His-CT584 in basic buffer [20 mM HEPES (pH 7.5), 50 mM KCl, 10 mM MgCl_2_, 1 mM dithiothreitol (DTT), 0.1% (v/v) Nonidet P-40] for 16 h at 4°C with gentle rocking. The two membranes were then processed by immunoblotting with anti-(6x)His and anti-GST antibodies, as appropriate.

## Results

### Screen for *C. trachomatis* Small and Acidic Proteins that Self-interact Identified Slc1, Mcsc, CT274, CT584, Scc4, CT790, and CT845

**Figure 1:**
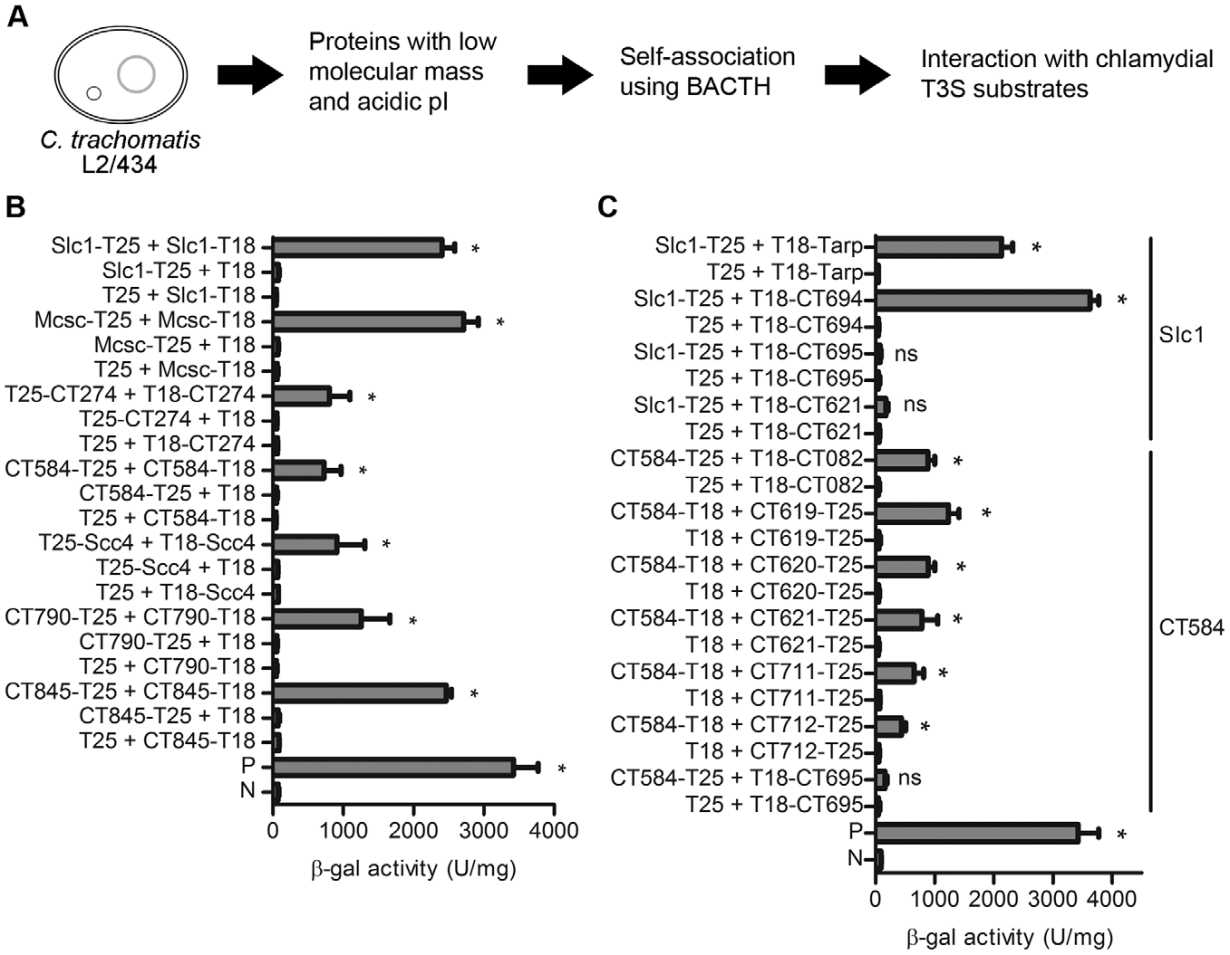
Bacterial two-hybrid screen for novel type III secretion (T3S) chaperone-substrate complexes of *C. trachomatis*. (A) The open reading frames within the genome of *C. trachomatis* L2/434 strain were analyzed for putative proteins with a predicted molecular mass of <30 kDa and isolectric point (pI) of <6 or with a molecular mass of 10–20 kDa and a pI of 6–7. We then selected those showing amino acid similarity to known T3S chaperones or with an unknown function. To determine if the 21 proteins thus identified could form dimers, we analyzed if they self-interacted using the bacterial adenylate cyclase two-hybrid (BACTH) system (B and data not shown). To determine if small and acidic proteins that can self-interact bind *C. trachomatis* T3S substrates, we systematically analyzed for protein-protein interactions using the BACTH) system (C). (B) and (C) β-galactosidase (β-gal) activity in *E. coli* BTH101 (*cya-99*) harboring plasmids encoding the indicated T18 and T25 fusion proteins. P, positive control: *E. coli* BTH101 expressing fusions of a leucine zipper protein to T18 and T25. N, negative control: *E. coli* BTH101 expressing T18 and T25. Data are the mean ± SEM from 3 independent experiments. Asterisks denote statistical significant differences relative to the negative control (P<0.05; student’s *t*-test).

The genome of *C. trachomatis* L2/434 is predicted to encode 889 proteins [47]. Among these putative proteins, we searched for those with predicted basic physico-chemical features of T3S chaperones (acidic and with a low molecular mass) (Fig. 1A). We excluded proteins with a predicted signal for the general secretory pathway, with predicted transmembrane domains, and whose deduced amino acid sequence indicated a function unrelated to a T3S chaperone. Given the evolutionary distance between *Chlamydiae* and Proteobacteria, we reasoned that primary and secondary structures of *C. trachomatis* T3S chaperones could have unique features. Based on this, we identified 21 *C. trachomatis* proteins as possible T3S chaperones: four previously identified chaperones (Slc1, Scc1, Mcsc, and Scc4) [29], [35], [36]; one protein with a tetratricopeptide motif [CT274; throughout this work we used the nomenclature of the annotated D/UW3 strain [48]], usually present in class II T3S chaperones [49]; one protein (CT584) that has been suggested to be the chlamydial needle tip protein [50], and whose ortholog in *C. pneumoniae* (Cpn0803) interacts with components of the injectisome [51]; and 15 proteins of unknown function (CT053, CT271, CT330, CT338, CT538, CT550, CT602, CT631, CT635, CT657, CT702, CT718, CT790, CT809, and CT845). We did not consider known class II or class III *C. trachomatis* chaperones. To assess whether these small and acidic proteins could form dimers, we analyzed if they self-interacted by using a bacterial two-hybrid system (BACTH) [43]. This system is based on restoring Cya activity to a Cya-deficient *E. coli*, by using the two fragments (T18 and T25) that constitute the catalytic domain of CyaA from *B. pertussis* fused to two potentially interacting proteins. Interaction between the two proteins fused to T18 or T25 reconstitutes activity of CyaA, which leads to an increase in cytosolic cyclic AMP levels and activation of the *lacZ* reporter gene. We therefore analyzed the activity of *lacZ*-encoded β-gal in bacteria expressing each of the selected 21 *C. trachomatis* proteins as T18 or T25 hybrid proteins (Fig. 1B and data not shown). The outcome of these experiments agreed with previous studies showing that Slc1, Mcsc, and CT584 oligomerize [29], [35], [50], [51] and revealed that CT274, Scc4, CT790, CT845 can self-interact (Fig. 1B).

### Systematic BACTH Protein-protein Interaction Assays Identified an Additional Putative Substrate of Slc1 and Six Possible Interacting Partners of CT584

To analyze if CT274, CT584, CT790, and CT845 could function as T3S chaperones, and whether Slc1, Mcsc, and Scc4 have additional interacting partners among *C. trachomatis* proteins, we performed a systematic analysis of protein-protein interactions by the BACTH system between these proteins and 17 previously identified *C. trachomatis* T3S substrates or effectors (CopN, CT119/IncA, CT223, CT225, CT288, CT233/IncC, Tarp, CT610/CADD, CT619, CT620, CT621, CT673/Pkn5, CT694, CT695, CT711, CT712, and CT847) [20]–[25], [52], [53], and also 18 newly identified *C. trachomatis* putative T3S substrates (C. Milho, M. Cunha, F. Almeida, R. Maurício, and L. J. Mota, unpublished data) (Fig. 1C and data not shown). Therefore, we tested protein-protein interactions by BACTH between 35 known or putative *C. trachomatis* T3S substrates and the 7 known or candidate chaperones (Mcsc, Slc1, Scc4, CT274, CT584, CT790, and CT845). These experiments confirmed that Slc1 interacts with Tarp and also revealed that it can bind CT694 (Fig. 1C). By the BACTH system, we were unable to detect an interaction of Slc1 with CT695, CT621, or with any other protein tested (Fig. 1C and data not shown). In addition, we observed that CT584 can interact with the five members (CT619, CT620, CT621, CT711, and CT712) of a family of chlamydial T3S substrates characterized by a domain of unknown function (DUF582 proteins) and with a newly identified *C. trachomatis* T3S substrate CT082 (see below), but not with CT695 or with any other protein tested (Fig. 1C and data not shown). We did not detect interactions of Mcsc, CT274, Scc4, CT790, or CT845 with any of the proteins tested (data not shown). This suggested that Slc1 can interact not only with Tarp but also with CT694, and that CT584 can interact with known (CT619, CT620, CT621, CT711, and CT712) and with a newly identified *C. trachomatis* T3S substrate (CT082; see below).

In the BACTH experiments described above we did not monitor expression of the proteins and therefore a negative result does not imply that two proteins do not interact. For example, by bacterial two-hybrid we did not detect the previously described binding of the Mcsc chaperone to Inc protein CT225 [29]. We analyzed full-length proteins, including Incs CT119/IncA, CT223, CT225, CT288, CT233/IncC, because we thought that a universal chaperone of Incs would bind to their characteristic hydrophobic region. However, because of the hydrophobic region, full-length Incs may aggregate. Furthermore, for practical reasons, we did not analyze all known Inc proteins or all possible topologies of T18 or T25 fusion proteins. Therefore, in addition to the interactions found, it is likely that other interactions were missed either because some of the fusion proteins may be unstable or because they were not tested.

### Slc1 Binds to the N-terminal Region of Tarp, CT694, and CT695

We next aimed to confirm the possible interactions between Slc1 and CT584 and its substrates by co-IP experiments and analyze their possible roles as T3S chaperones, by using *Y. enterocolitica* as a heterologous host. We describe first the studies done with Slc1.

**Figure 2:**
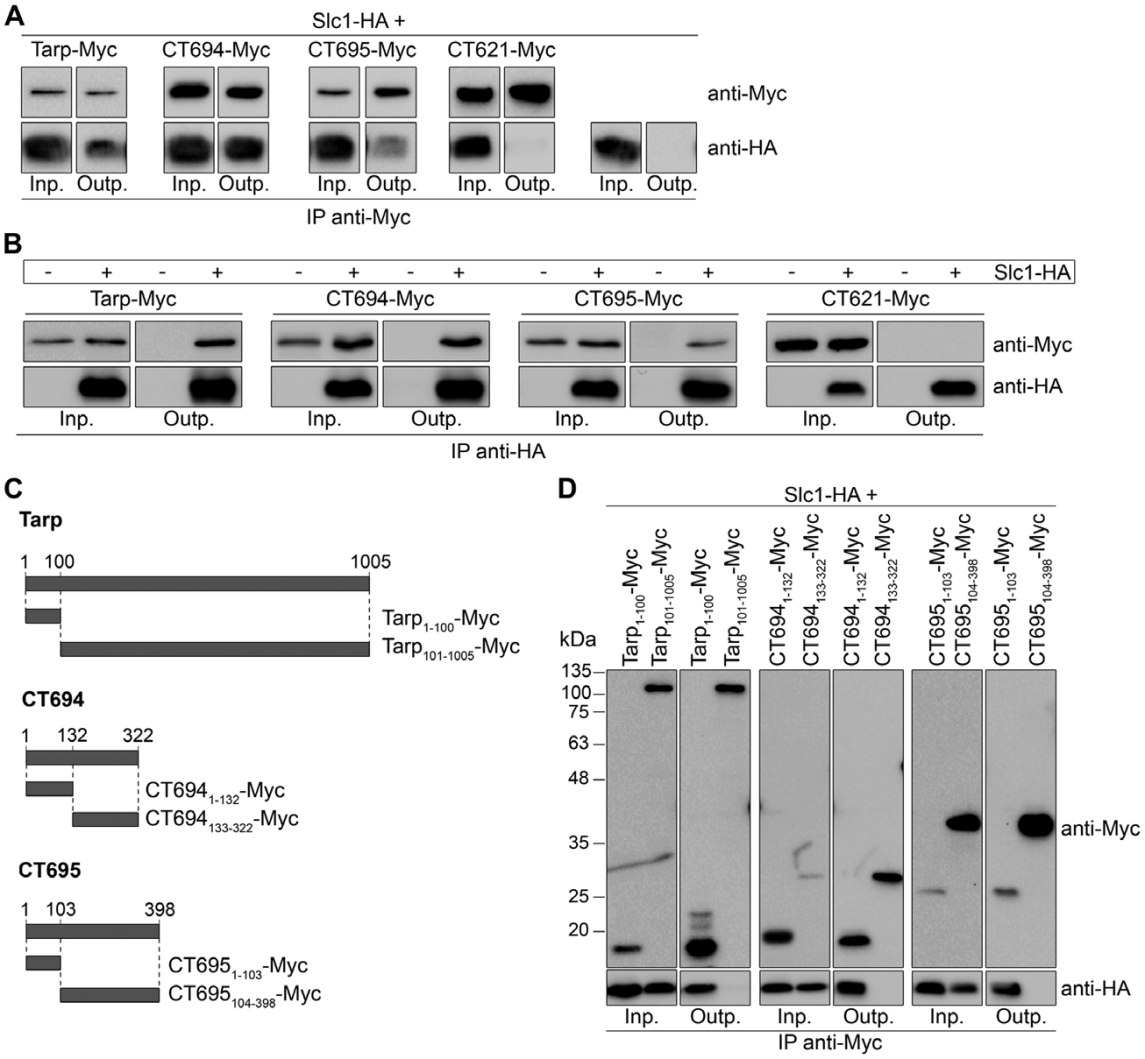
Slc1 interacts with the N-terminal region of Tarp, CT694, and CT695. (A, B, and D) *Y. enterocolitica* ΔHOPEMT strains expressing the indicated proteins were grown in non-secreting conditions. The bacterial cells were lysed and proteins in the lysate supernatants (input) were immunoprecipitated with mouse monoclonal anti-Myc (A and D) or anti-HA (B) antibodies bound to Protein G agarose beads (output). The input (Inp.) and output (Outp.) fractions from the immunoprecipitations (IPs) were analyzed by immunoblotting with rabbit polyclonal anti-Myc and rat monoclonal anti-HA antibodies. (C) Scheme of the truncated Myc-tagged Tarp, CT694, and CT695 proteins analyzed to determine the binding regions of Slc1.

We analyzed the possible Slc1-CT694 complex, using as reference the described interaction between Slc1 and Tarp [35]. As CT695 has been previously shown to be a T3S substrate [25], which is encoded by a gene possibly in the same transcriptional unit as *ct694* [25], we suspected that our inability to detect a Slc1-CT695 interaction by BACTH (Fig. 1C) could be due to topological constraints of the T18 and T25 fusion proteins used. Therefore, we also analyzed a possible interaction between Slc1 and CT695 by co-IP. In addition, we analyzed the interaction between Slc1 and CT621 as a negative control, as these two proteins did not interact when using the BACTH system (Fig. 1C). We used T3S-proficient *Y. enterocolitica* ΔHOPEMT strains harboring plasmids expressing the relevant proteins and grown in non-secreting conditions (in the presence 5 mM Ca^2+^). A mouse monoclonal anti-Myc antibody was used to pull-down proteins from extracts of ΔHOPEMT co-expressing Tarp, CT694, or CT695 with a C-terminal Myc tag (Tarp-Myc, CT694-Myc, or CT695-Myc) and Slc1 with a C-terminal HA tag (Slc1-HA). This showed that the IP of Tarp-, CT694-, or CT695-Myc pulled down Slc1-HA (Fig. 2A and Fig. S1A). In parallel, we performed similar experiments in which Slc1-HA was expressed alone or co-expressed with C-terminally Myc-tagged CT621 (CT621-Myc). In these conditions, Slc1-HA was not pulled down by the mouse anti-Myc antibody (Fig. 2A and Fig. S1A). Furthermore, in reverse experiments using a mouse monoclonal anti-HA antibody to IP Slc1-HA, we pulled down Tarp-, CT694-, and CT695-Myc, but not CT621-Myc (Fig. 2B). We also detected the interaction between CT043 and Tarp, CT694, or CT695 by co-IP when the *Y. enterocolitica* strains were grown in T3S-inducing conditions (Fig. S2A). By co-IP, we did not detect an interaction between Slc1 and CT696 (data not shown), which encodes a gene that may also be co-transcribed with *ct694* and *ct695* [25].

T3S chaperones of effectors (class I chaperones) normally bind to the N-terminal region of their substrates [1], [3], and Slc1 binds to the first 200 amino acids of Tarp [35]. To analyze the binding region of Slc1 within its three substrates, we constructed plasmids encoding truncated versions of Tarp, CT694, and CT695 with a C-terminal Myc-tag, as depicted in Fig. 2C. These proteins were individually co-expressed with Slc1-HA in *Y. enterocolitica* ΔHOPEMT and co-IP experiments using an anti-Myc antibody were performed as described above. This showed that the first 100, 132, or 103 amino acids of Tarp, CT694, or CT695, respectively, were necessary and sufficient for binding of Slc1 (Fig. 2D). For unknown reasons, the migration of one of the Myc-tagged truncated versions of CT695 (CT695_1–103_-Myc; Fig. 2C) on SDS-PAGE was slower than expected from its predicted molecular mass (∼14 kDa) (Fig. 2D). In summary, Slc1 interacts with the N-terminal region of Tarp, CT694, and CT695.

### Slc1 Promotes T3S of Tarp, CT694, and CT695 in *Y. enterocolitica*

**Figure 3:**
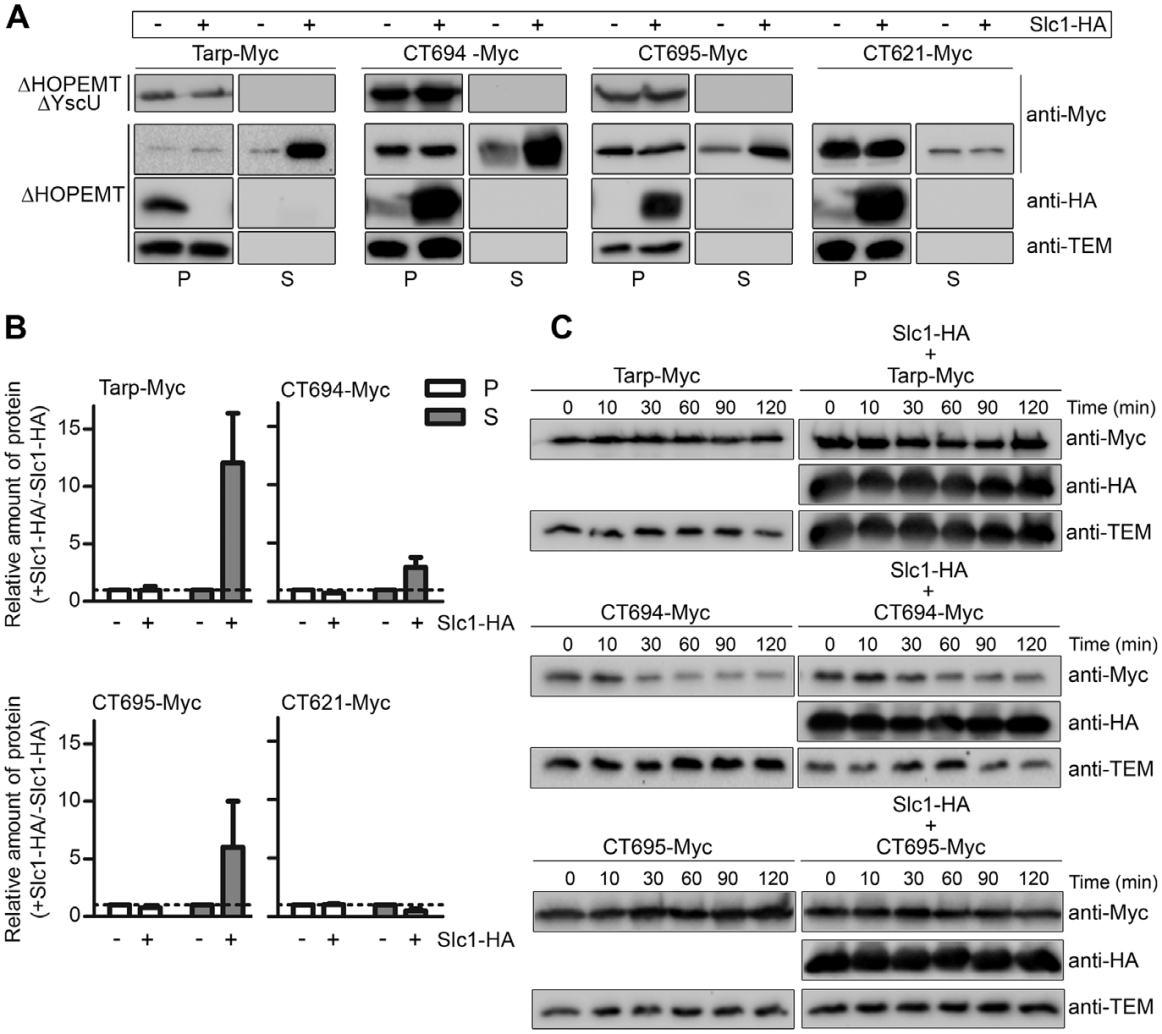
Slc1 promotes type III secretion (T3S) of Tarp, CT694, and CT695 in *Y. enterocolitica*. (A) T3S-deficient *Y. enterocolitica* ΔHOPEMT ΔYscU and T3S-proficient ΔHOPEMT strains expressing the indicated proteins were incubated in T3S-inducing conditions [42]. Proteins in culture supernatants (S - secreted proteins), and in bacterial pellets (P - non-secreted proteins) from ∼5×10^8^ and ∼5×10^7^ bacteria, respectively, were analyzed by immunoblotting with the indicated antibodies. Immunodetection of TEM-1 β-lactamase (encoded by pBAD/Myc-His–derived plasmids expressing Myc-tagged proteins) ensured that presence of proteins in culture supernatants was not a result of bacterial lysis or contamination. (B) The amount of protein in bacterial pellets and in secreted fractions of assays done with ΔHOPEMT-derived strains was estimated by densitometry analyses of immunoblot images. We calculated the ratio between the amounts of each protein (Tarp-Myc, CT694-Myc, CT695-Myc or CT621-Myc) in the presence of Slc1-HA relative to when the proteins were expressed alone (+ Slc1-HA/− Slc1-HA). The dashed line indicates a ratio of 1. Data are the mean ± SEM from 3 independent experiments. (C) *Y. enterocolitica* ΔHOPEMT strains expressing the indicated proteins were grown in non-secreting conditions (see Materials and Methods). Chloramphenicol was added (time = 0 min) to stop bacterial protein synthesis. Samples were then taken at the depicted time points and analyzed by immunoblotting with the indicated antibodies.

**Figure 4:**
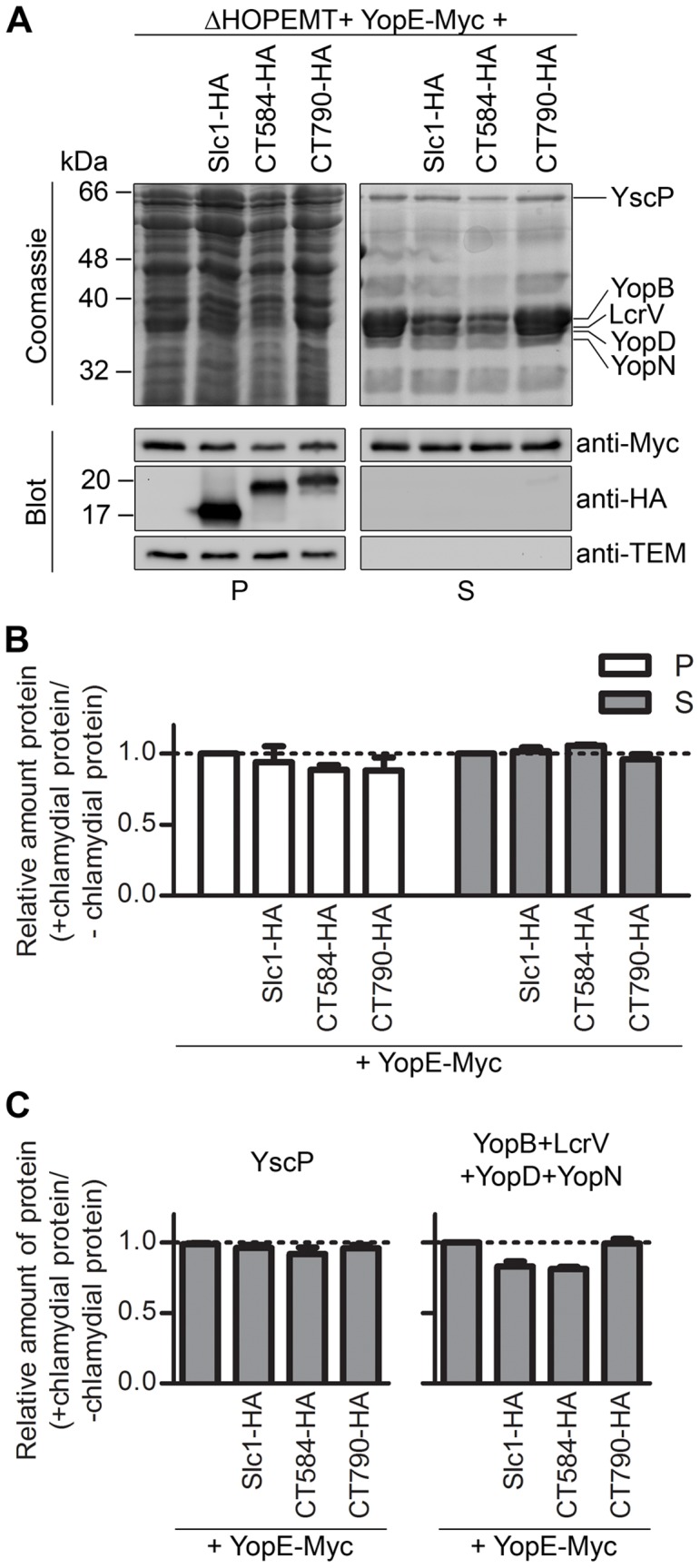
Effect of the expression of Slc1 or CT584 on *Yersinia* type III secretion (T3S). (A) *Y. enterocolitica* ΔHOPEMT carrying plasmids encoding Slc1-HA, CT584-HA or CT790-HA, and YopE-Myc (as indicated) were incubated in T3S-inducing conditions [42]. Proteins in culture supernatants (S - secreted proteins), and in bacterial pellets (P - non-secreted proteins) were analyzed by SDS-PAGE followed by Coomassie staining and immunoblotting with the indicated antibodies. Immunodetection of TEM-1 β-lactamase (encoded by the plasmid expressing YopE-Myc, a pBAD-Myc-His–derivative) ensured that presence of proteins in culture supernatants was not a result of bacterial lysis or contamination. Proteins from culture supernatants corresponding to ∼5×10^8^ or ∼2.5×10^8^ bacteria and from bacterial pellets corresponding to ∼5×10^7^ or ∼2.5×10^7^ bacteria were analyzed by Coomassie staining or by immunoblotting, respectively. (B) The amount of YopE-Myc in the bacterial pellets and in the secreted fractions was analyzed by densitometry from images of immunoblots. (C) The amount of YscP (graph on the left) and of YopB, YopD, LcrV and YopN (graph on the right) in secreted fractions was analyzed by densitometry from images of Coomassie-stained gels. YopB, YopD, LcrV, and YopN were analyzed all together because single bands corresponding to these proteins could not be easily distinguished in Coomassie-stained gels. The bands corresponding to YopB, YopD, LcrV, YopN, or YscP were deduced from the well known separation on SDS-PAGE of *Yersinia* secreted proteins after a T3S assay [42]. In (B) and (C), we calculated the ratio between the amounts of YopE-Myc, YscP, or YopB, YopD, LcrV, and YopN when they were expressed in the presence of chlamydial proteins (Slc1-HA, CT584-HA, or CT790-HA) relative to when they were expressed alone (+ chlamydial proteins/− chlamydial proteins). All data are the mean ± SEM from 3 independent experiments.

To address the possible role of Slc1 as a chaperone, we analyzed if it could assist T3S of Tarp, CT694 and CT695. For this, we used T3S-proficient (ΔHOPEMT) or T3S-deficient (ΔHOPEMT ΔYscU) *Y. enterocolitica* carrying plasmids encoding Tarp-Myc, CT694-Myc, or CT695-Myc (described above in the co-IP experiments). T3S of these proteins was analyzed when they were expressed alone or co-expressed with Slc1-HA. Bacterial strains were incubated in T3S-inducing conditions (without Ca^2+^) [42], followed by fractionation into culture supernatants and bacterial pellets. The plasmids expressing Myc-tagged proteins are derivatives of pBAD/*Myc*-His A (Invitrogen), encoding TEM-1 β-lactamase. Immunodetection of TEM-1 only in the bacterial pellet fraction controlled that detection of proteins in the supernatant fraction did not result from bacterial lysis or contamination with the bacterial pellet (Fig. 3A). Immunoblotting analyses of the proteins in the two fractions revealed T3S of Tarp-, CT694-, and CT695-Myc in the absence or presence of Slc1-HA (Fig. 3A). However, the amount of the proteins in the culture supernatant was consistently higher when they were co-expressed with Slc1-HA (Fig. 3A and B). Specifically, quantification of the immunoblot images by densitometry revealed that the ratio of the amounts of Tarp-Myc, CT694-Myc, or CT695-Myc in culture supernatants when they were co-expressed with Slc1-HA relative to when they were expressed alone was 12±4, 3±1, or 6±4 (average ± SEM from three independent experiments), respectively (Fig. 3B). In parallel experiments, the amount of secreted CT621-Myc was slightly reduced by co-expression of Slc1-HA (ratio of 0.5±0.2; Fig. 3A and B). As expected from its chaperone role, Slc1 was not secreted by *Y. enterocolitica* (Fig. 3A). The small reduction in secretion of CT621-HA was similar to the effect of the expression of Slc1-HA on the secretion of specific *Yersinia* T3S substrates (Fig. 4). However, T3S of the *Yersinia* effector YopE was not affected by the expression of Slc1-HA (Fig. 4). In contrast, expression of Slc1-HA specifically increased the amount of secreted Tarp-Myc, CT694-Myc, and CT695-Myc by *Y. enterocolitica* (Fig. 3A and B). Overall, this indicated that Slc1 is a chaperone with multiple substrates.

To obtain insights on the mechanism of action of Slc1, we analyzed if its expression affected the intra-bacterial stability of Tarp, CT694 or CT695. *Y. enterocolitica* ΔHOPEMT co-expressing Slc1-HA and Tarp-Myc, CT694-Myc, or CT695-Myc, or expressing Tarp-Myc, CT694-Myc, or CT695-Myc alone, were grown in non-secreting conditions allowing for protein expression. Chloramphenicol was then added to stop bacterial protein synthesis and samples were periodically taken in the subsequent 120 min. The protein levels were then analyzed by immunoblotting with anti-Myc and anti-HA antibodies, as well as with anti-TEM-1 antibodies (as a loading control). This revealed that the intra-bacterial stability of Tarp-, CT694-, and CT695-Myc was not significantly affected by the presence of Slc1-HA (Fig. 3C).

### CT584 Binds to a 120-amino Acids Residue Central Region of CT082

**Figure 5:**
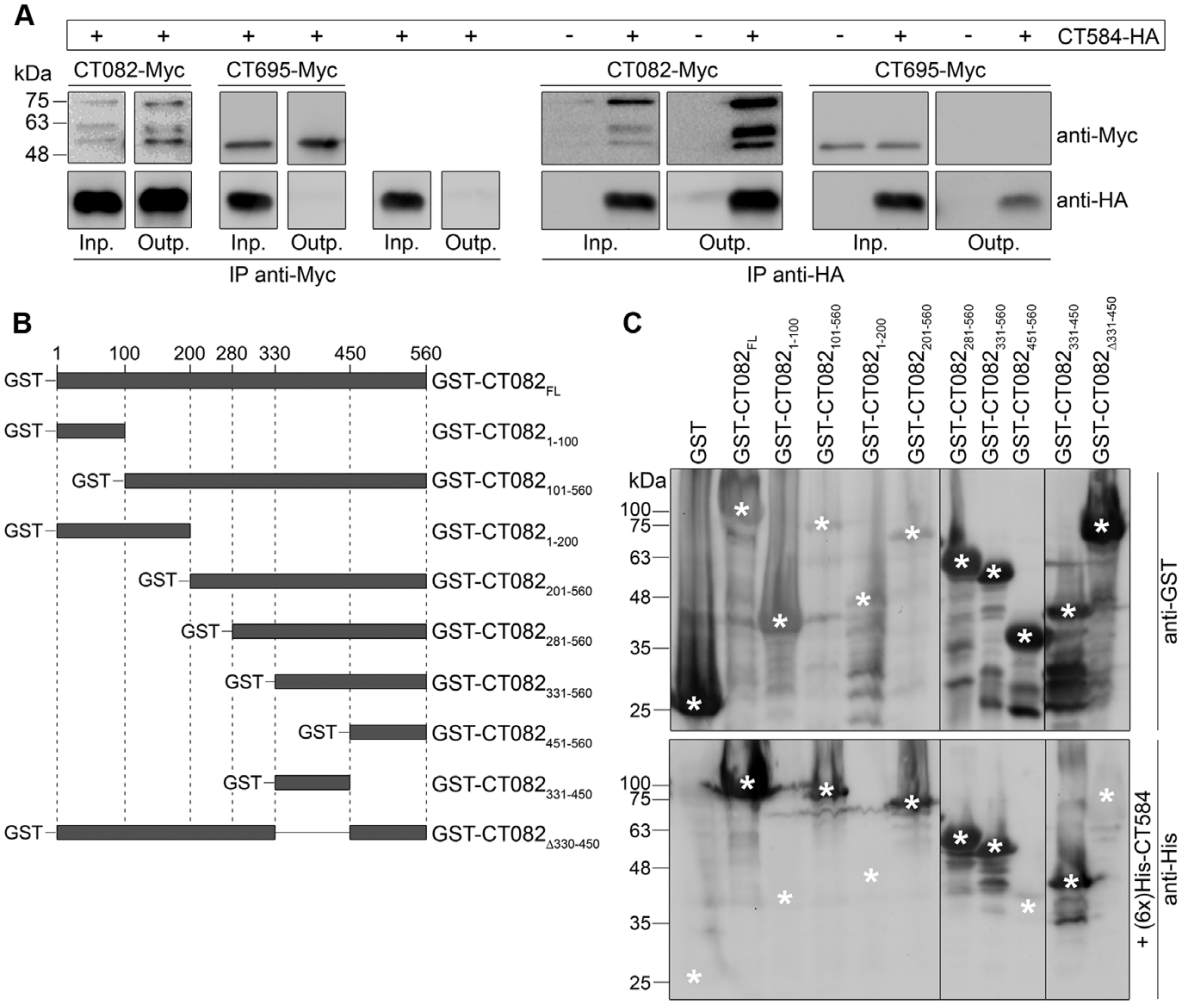
CT584 interacts with a central region of CT082. (A) *Y. enterocolitica* ΔHOPEMT strains expressing the indicated proteins were grown in non-secreting conditions. The bacterial cells were lysed and proteins in the lysate supernatants (input) were immunoprecipitated with mouse monoclonal anti-Myc or anti-HA antibodies (as depicted) bound to Protein G agarose beads (output). The input (Inp.) and output (Outp.) fractions from the immunoprecipitations (IPs) were analyzed by immunoblotting with rabbit polyclonal anti-Myc and rat monoclonal anti-HA antibodies. (B) Scheme of the truncated GST-tagged CT082 proteins analyzed to determine the binding region of CT584. (C) Protein overlay binding assays to identify the binding region of CT584 within CT082. Two identical SDS-PAGE were loaded with extracts of *E. coli* expressing the indicated GST fusion proteins. The electrophoresed proteins were transferred onto nitrocellulose membranes. One membrane was immunodetected with anti-GST antibodies while the other was probed with purified (6x)His-CT584 before being immunodetected with anti-His antibodies. Asterisks indicate the position corresponding to the predicted migration on SDS-PAGE of full-length GST fusion proteins (upper image) and the predicted position where an anti-His-dependent signal should appear if (6x)His-CT584 would bind the GST fusion proteins (lower image).

We were unable to confirm the interaction between CT584 and the DUF582 proteins (CT619, CT620, CT621, CT711, and CT712) using co-IP, GST pull-downs, co-purifications, or overlay assays (data not shown). Because appropriate internal controls were used in these experiments (not shown), this suggests that the interaction between CT584 and DUF proteins observed by BACTH was probably an artifact. On the other hand, a protein overlay binding assay using purified (6x)His-CT584 to probe extracts of *E. coli* expressing either GST alone or a fusion of GST to the N-terminus of CT082 (GST-CT082) provided a first confirmation of the interaction between CT584 and CT082 (Fig. 5C). In addition, co-IP experiments using a monoclonal anti-Myc antibody to pull down proteins from extracts of *Y. enterocolitica* ΔHOPEMT grown in non-secreting conditions and co-expressing C-terminally Myc-tagged CT082 (CT082-Myc) and C-terminally HA-tagged CT584 (CT584-HA), showed co-IP of CT584-HA (Fig. 5A and Fig. S1B). CT584-HA was not co-immunoprecipitated after similar experiments in which it was expressed alone or co-expressed with CT695-Myc (Fig. 5A and Fig. S1B) (CT695 did not show an interaction with CT584 by the BACTH system; Fig. 1C). In reverse co-IPs using a mouse monoclonal anti-HA antibody to IP CT584-HA, we observed pull down of CT082-Myc but not of CT695-Myc (Fig. 5A). Furthermore, identical results were obtained when *Y. enterocolitica* strains were grown in T3S-inducing conditions (Fig. S2B). Overall, these experiments showed that CT584 interacts with CT082.

Different bands were observed on the immunoblots from extracts of bacteria expressing CT082-Myc: a band with an apparent molecular mass on SDS-PAGE of ∼75 kDa, and two other bands of ∼62 and ∼57 kDa (Fig. 5A). Full-length CT082-Myc has a predicted molecular mass of ∼63 kDa, and the most likely explanation for the different bands seen is that CT082-Myc migrates on SDS-PAGE slower than expected (corresponding to the ∼75 kDa band) and that the faster migrating bands are degradation products. CT082 was also apparently unstable when expressed in *E. coli* as a (6x)-His- or a GST-tagged protein, as suggested by the appearance on SDS-PAGE of multiple putative proteolytic fragments (Fig. 5C and data not shown).

To analyze the binding region of CT584 within CT082, we first tested if CT584 would bind the N-terminal part of its substrate. For this, we constructed plasmids encoding relevant truncated versions of CT082 fused to GST (GST-CT082_1–100_, GST-CT082_101–560_, GST-CT082_1–200_ and GST-CT082_201–560_; Fig. 5B). The proteins were expressed in *E. coli* and an overlay binding assay using (6x)His-CT584 revealed interactions only with GST-CT082_101–560_ and GST-CT082_201–560_, but not with GST-CT082_1–100_ or GST-CT082_1–200_. This indicated that the interacting region of CT584 within CT082 should lay between amino acid residues 201 and 560 (Fig. 5C). To further map this region, we constructed plasmids encoding GST-CT082_281–560_, GST-CT082_331–560_, GST-CT082_451–560_, GST-CT082_330–450_, and GST-CT082_Δ330–450_ (Fig. 5B). Protein overlay binding assays with (6x)His-CT584 showed that the amino acid residues 330 to 450 of CT082 were necessary and sufficient for binding of CT584 (Fig. 5C).

### CT584 Increases the Amount of Type III Secreted CT082 by Promoting its Intra-bacterial Stability in *Y. enterocolitica*

**Figure 6:**
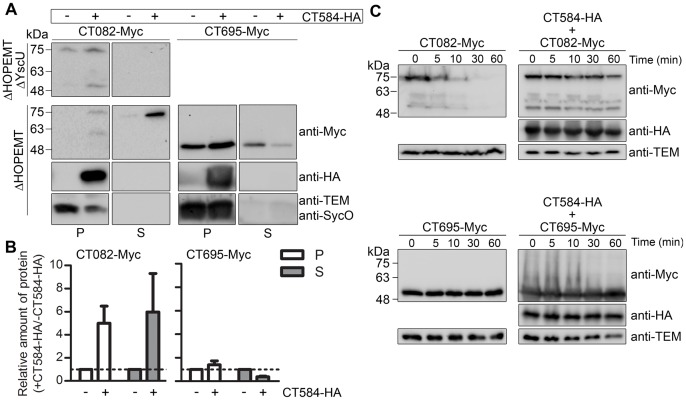
CT584 promotes type III secretion (T3S) and intra-bacterial stability of CT082 in *Y. enterocolitica*. T3S-deficient *Y. enterocolitica* ΔHOPEMT ΔYscU and T3S-proficient *Y. enterocolitica* ΔHOPEMT carrying plasmids encoding Myc-tagged CT082 or CT695, and HA-tagged CT584 (as indicated) were incubated in T3S-inducing conditions [42]. Proteins in culture supernatants (S - secreted proteins), and in bacterial pellets (P - non-secreted proteins) from ∼5×10^8^ and ∼5×10^7^ bacteria, respectively, were analyzed by immunoblotting with anti-Myc, anti-HA, and anti-TEM or anti-SycO antibodies. Immunodetection of TEM-1 β-lactamase (encoded by pBAD/Myc-His–derived expressing Myc-tagged proteins) or the *Y. enterocolitica* T3S chaperone SycO [4] in the CT082-Myc or CT695-Myc T3S assays, respectively, ensured that presence of proteins in culture supernatants was not a result of bacterial lysis or contamination. (B) The amount of protein in bacterial pellets and in secreted fractions of assays done with ΔHOPEMT-derived strains estimated by densitometry analyses of immunoblot images. We calculated the ratio between the amounts of each protein (CT082-Myc or CT695-Myc) in the presence of CT584-HA relative to when the proteins were expressed alone (+ CT584-HA/− CT584-HA). The dashed line indicates a ratio of 1. Data are the mean ± SEM from 4 independent experiments. (C) *Y. enterocolitica* ΔHOPEMT carrying plasmids encoding Myc-tagged CT082 or CT695, and HA-tagged Slc1 (as indicated) were grown in non-secreting conditions (see Materials and Methods). Chloramphenicol was added (time = 0 min) to stop bacterial protein synthesis and samples were taken at the indicated time points. Samples were analyzed by immunoblotting using anti-Myc, anti-HA, and anti-TEM antibodies.

To analyze the possible role of CT584 as chaperone of CT082, we tested if CT584 could assist T3S of CT082, using *Y. enterocolitica* as a heterologous host. We used the plasmids encoding CT584-HA and CT082-Myc, described above in the co-IP experiments (Fig. 5A), and the T3S assays were done as detailed for Slc1 (Fig. 3A). We detected T3S of CT082-Myc in the absence or presence of CT584-HA (Fig. 6A), which showed that CT082 is a *C. trachomatis* T3S substrate. However, the amount of the protein in the culture supernatant was consistently higher when it was co-expressed with CT584 (Fig. 6A and B). Specifically, quantification of the immunoblot images by densitometry revealed that the ratio of the amounts of CT082-Myc in culture supernatant when it was co-expressed with CT584-HA relative to when it was expressed alone was 6±3 (average ± SEM from four independent experiments). This effect was specific, as in parallel experiments the amount of secreted CT695-Myc was slightly reduced by the co-expression of CT584-HA (ratio of 0.4±0.1; Fig. 6A and B). As observed with Slc1-HA, expression of CT584-HA slightly affected secretion of specific *Yersinia* T3S substrates, but not of the YopE effector (Fig. 4). In the CT695-Myc T3S assays, we used antibodies against the *Y. enterocolitica* endogenous T3S chaperone SycO [4] to control that detection of proteins in the supernatant fraction did not result from bacterial lysis or contamination with the bacterial pellet (Fig. 6A). In the case of CT082-Myc T3S assays, we controlled this with anti-TEM-1 antibodies (Fig. 6A), as previously explained for Slc1 (Fig. 3A). We did not detect secretion of CT584-HA (Fig. 6A), suggesting that CT584 is not a T3S substrate. Overall, these experiments indicated that CT584 can function as a T3S chaperone of CT082.

To obtain insights on the mechanism by which CT584 could exert its chaperone function, we analyzed if its expression affected the intra-bacterial stability of CT082, using *Y. enterocolitica* co-expressing CT584-HA and CT082-Myc or CT695-Myc, or CT082-Myc or CT695-Myc alone, as described above for Slc1. This revealed that the intra-bacterial stability of CT082-Myc, but not of CT695-Myc, was enhanced by the presence of CT584-HA (Fig. 6C). Therefore, CT584 may function by promoting the intra-bacterial stability of CT082 before secretion, although this does not rule out other possibilities such as a role of CT584 in targeting CT082 for secretion.

### CT082 and CT584 are Expressed during the Developmental Cycle of *C. trachomatis*

In previous studies, the expression of Tarp [24], [35], CT694 [25], and Slc1 [35] was detected by immunoblotting and/or immunofluorescence during the developmental cycle of *C. trachomatis*. Furthermore, recent quantitative proteomics revealed the presence of Tarp, CT082, CT694, CT695, Slc1 and CT584 on *C. trachomatis* EBs, and of Slc1 and CT584 on *C. trachomatis* RBs [54].

To further support that CT082 and CT584 are expressed during the *C. trachomatis* developmental cycle, we generated rabbit polyclonal antibodies against these two proteins. The antibodies specifically recognized a fusion of enhanced GFP (EGFP) to the N-terminus of CT082 (EGFP-CT082) (Fig. S3A) and a C-terminally HA-tagged CT584 (CT584-HA) (Fig. S3B), respectively, after their ectopic expression in HeLa cells. The anti-CT082 and anti-CT584 antibodies did not recognize ectopically expressed EGFP (Fig. S3A) and CT082-HA (Fig. S3B), respectively. To analyze expression of CT082 and CT584 during the developmental cycle of *C. trachomatis*, we infected HeLa 229 cells with the L2/434 strain for 2, 8, 14, 20, 26, 32, 38, or 44 h. By immunoblotting of whole lysates of infected cells, we were able to detect expression of CT584 from 14 h post-infection (Fig. S3D) and of CT082 from 26 h post-infection (Fig. S3C). In further agreement with the previous quantitative proteomics data [54], we could detect CT584 in EBs and RBs and CT082 only in EBs (data not shown). As observed before in *Yersinia* protein extracts (Fig. 5 and 6), CT082 migrated on SDS-PAGE with an apparent molecular mass (∼75 kDa) higher than expected from its predicted molecular mass (60 kDa). Analysis of extracts of mammalian cell ectopically expressing CT082-HA (predicted molecular mass of 61 kDa) by immunoblotting also revealed abnormal migration on SDS-PAGE of this protein (Fig. S3A and B). Putative degradation products of ectopically or endogenously expressed CT082 (Fig. S3B and not shown) were also occasionally seen. Migration of EGFP-CT082 (predicted molecular mass of 86 kDa) on SDS-PAGE may also be slower than expected (Fig. S3A), but the limited resolution in this upper region of the gel does not allow a clear conclusion.

In summary, in agreement with previous quantitative proteomics data [54], and as shown before for Tarp [24], [35], Slc1 [35], and CT694 [25], these results indicate that CT082 and CT584 are expressed during the developmental cycle of *C. trachomatis*. CT695 remains to be immunodetected during the chlamydial developmental cycle; however, as mentioned above, quantitative proteomics clearly detected CT695 on EBs [54], which strongly supports that this protein is also expressed by *C. trachomatis*.

### Expression of Slc1 and CT584 in *Y. enterocolitica* does not Affect Secretion of the *Yersinia* T3S Effector YopE

A possible alternative explanation for the results described above would be that expression of Slc1-HA or CT584-HA caused a general up-regulation of T3S by *Y. enterocolitica*. We analyzed this possibility, although, if anything, expression of Slc1-HA or CT584-HA slightly reduced secretion by *Yersinia* of chlamydial T3S substrates (CT621 or CT695, respectively) that are not their binding partners (Fig. 3 and Fig. 6). We performed T3S assays with strain ΔHOPEMT expressing the *Yersinia* effector YopE with a C-terminal Myc-tag (YopE-Myc), in the presence or absence of Slc1-HA or CT584-HA. YopE-Myc was expressed under the control of arabinose-inducible *P_BAD_*, as all chlamydial T3S substrates examined in this work. As control, we also analyzed the consequences of expressing the *Chlamydia* protein CT790 with a C-terminal HA tag (CT790-HA) on *Yersinia* T3S. In BACTH assays, CT790 showed to self-interact (as Slc1 and CT584) but did not bind to any of the *C. trachomatis* T3S substrates tested (Fig. 1 and data not shown). *Y. enterocolitica* ΔHOPEMT strains expressing YopE-Myc alone or in the presence of Slc1-HA, CT584-HA, or CT790-HA were incubated in T3S inducing conditions. After that, proteins in culture supernatants and in bacterial pellets were analyzed by SDS-PAGE and immunoblotting (to monitor secretion of YopE-Myc) or Coomassie staining (to monitor secretion of endogenous *Y. enterocolitica* T3S substrates encoded by strain ΔHOPEMT). This showed that expression of Slc1-HA, CT584-HA or CT790-HA did not affect the amount of secreted YopE-Myc (Fig. 4A and 4B; please compare with Fig. 3B or Fig. 6B). Expression of Slc1-HA, CT584-HA or CT790-HA also did not affect secretion of YscP (Fig. 4A and 4C), a *Yersinia* T3S substrate that controls the length of the needle of the injectisome [55]. On the other hand, expression of Slc1-HA or CT584-HA, but not of CT790-HA, caused a slight but reproducible negative effect on secretion of the *Yersinia* T3S translocators (YopB, YopD, and LcrV) and of a *Yersinia* T3S regulator (YopN) (Fig. 4A and 4C). The amounts of secreted YscP, YopB, YopD, LcrV and YopN were analyzed from Coomassie stained gels based on the well known separation on SDS-PAGE of *Yersinia* secreted proteins after a T3S assay [42]. The amounts of YopB, YopD, LcrV and YopN in the secreted fraction were analyzed all together because a defined band for each of these four proteins could not be easily distinguished on Coomassie-stained gels. We do not know the exact reason why expression of Slc1 or CT584 in ΔHOPEMT affected secretion of YopB, YopD, YopN, and LcrV, but not of YopE-Myc or of YscP. However, T3S chaperones need to interact with the injectisome to release their substrates before secretion [2], [56], and therefore Slc1 and CT584 may interact with components of the *Yersinia* injectisome to help T3S of their substrates (Tarp, CT694, CT695, and CT082). Considering that Slc1 and CT584 did not evolve in *Yersinia*, their possible interaction with components of the *Y. enterocolitica* injectisome may also hamper the normal functioning of this T3SS. Therefore, these analyses supported the idea that Slc1 and CT584 are T3S chaperones.

## Discussion

The *C. trachomatis* T3S system likely mediates bacterial uptake, intracellular survival and proliferation during infection [30]. However, relatively little is known about the identity and function of its different elements. In this work, we identified Slc1 as a chaperone of multiple T3S substrates (Tarp, CT694, and CT695) and CT584 as a new chaperone for a newly identified T3S substrate (CT082). In the initial BACTH screen, additional binding partners of Slc1 or CT584 (or of other known and putative chaperones) may have escaped unnoticed among the T3S substrates tested. For example, by bacterial two-hybrid, we did not detect the Slc1-CT695 interaction. As mentioned before, we did not monitor expression of the fusion proteins used in the BACTH assay and we did not test all possible topologies of T18 or T25 fusion proteins. It is also possible that Slc1 and/or CT584 may have additional binding partners that we did not test in this study.

It is well-established that, in addition to effectors, T3SSs also secrete components of the injectisome [57], translocon and needle tip or filament proteins [58], [59], and regulatory proteins [60]–[63], and that all these different types of T3S substrates can have chaperones [1], [3], [10]. Thus, a T3S substrate is not necessarily an effector. Tarp and CT694 are known *Chlamydia* effectors, i.e., it has been shown that they are translocated across the inclusion membrane in infected cells and interfere with host cell functions [24], [25], but the same has not yet been shown for CT082 or CT695. As will be discussed below, the characteristics of the interaction between Slc1 and its substrates indicate that CT695 is an effector, while those of the CT584-CT082 complex suggest that CT082 is a T3S substrate other than an effector.

Effector T3S chaperones (class I chaperones) normally bind to the N-terminal region of their substrates and are distinguished according to whether they have one (class IA) or several substrates (class IB) [2], [10]. Slc1 is at least the chaperone of two known chlamydial effectors (Tarp and CT694), and of one chlamydial T3S substrate (CT695), and binds to their N-terminal regions. This indicates that Slc1 is a multi-effector chaperone (class IB) and therefore supports that CT695 is an effector. Inspection of the binding regions of Slc1 within Tarp, CT694, and CT695, did not reveal a conserved sequence or motif common to the three proteins, as had been observed before for other class IB chaperones [9], [64]. In fact, a conserved structural motif (the β-motif) has been found in effectors that interact with class IA and class IB chaperones [9], and an amino acid sequence [(LMIF)_1_XXX(IV)_5_XX(IV)_8_X(N)_10_] has been identified as a “conserved chaperone-binding domain” (CCBD) recognized by class IB chaperones [64]. In particular, the CCBD sequence is apparently widespread as it is recognized by class IB chaperones from different human pathogens (*Proteus mirabilis*, *Burkholderia dolosa*, *Y. enterocolitica*, *Salmonella*, and *Shigella*) as well as from endosymbionts (*Sodalis glossinidius* and *Hamiltonella defensa*) [64]. A CCBD sequence is not present within the binding region of Slc1 in Tarp, CT694 or CT695. It unknown why Slc1 does not function by recognizing this CCBD sequence, but this may relate to the evolutionary distance between the T3SS of *Chlamydia* and of those of Proteobacteria.

Regarding the mode of action of Slc1, Slc1-substrate complexes could act as a 3D T3S signal [7], or Slc1 could maintain their substrates in a conformation favoring T3S [5]. In addition, CT694 has a membrane localization domain (MLD) between amino acid residues 40–80 [65], which is within the binding region of Slc1. These MLDs are found in several T3S effectors, which need to localize to host cell membranes for their function [65]. It is possible that this MLD within CT694 is detrimental for T3S unless it is covered by Slc1 [4]. Regarding the other known binding substrates of Slc1, while Tarp does not seem to significantly associate with host cell membranes during infection [65], it is unknown whether CT695 associate with membranes.

Tarp and CT694 are immediate early effectors, stored in EBs and likely involved in the invasion-associated re-organization of the host cell cytoskeleton [24], [25], [66], [67]. Considering that the *ct695* gene is possibly co-transcribed with *ct694* [25], that CT695 is abundantly present in EBs [54], and the characteristics of the Slc1-CT695 interaction, it is likely that CT695 is also an immediate early effector. Therefore, we speculate that Slc1 may have an important role controlling the delivery of effectors at the early stages of *C. trachomatis* infection.

Orthologs of CT584 and CT082 can be found in sequenced genomes of *Chlamydiaceae*, but not in those of other members of the *Chlamydiae* phylum. Their amino acid sequences do not show significant similarity to any other proteins in the databases. Furthermore, the 3D structure of Cpn0803, the ortholog of CT584 in *C. pneumoniae* (Cpn0803 and CT584 display 83% of identity and 92% of similarity), does not significantly resemble other proteins in structural databases [51]. A biophysical characterization of CT584 had suggested it could be the chlamydial needle tip protein [50]. However, the subsequent structural characterization of Cpn0803 did not support such hypothesis [51].

CT584 binds CT082 (Fig. 5) and promotes its T3S by *Y. enterocolitica* probably by stabilizing CT082 in the cytosol prior to secretion (Fig. 6). These results indicated that CT584 acts as a T3S chaperone of CT082. Indirect evidence that CT584 could function as a chaperone were previously described interactions between its ortholog in *C. pneumoniae* (Cpn0803) and chlamydial injectisome proteins CdsN and CdsQ [51]. Homologs of CdsN and CdsQ are present in all T3S systems [1], and different T3S chaperones have been shown to interact with them [2]. However, Cpn0803 also interacts with the chlamydial needle protein (CdsF) [51]. This is harder to explain for a T3S chaperone, but CT584 could have additional functions, which is not unusual for T3S chaperones [2], [10].

The characteristics of CT584 and of its binding to CT082 suggest that it is not a class I chaperone. This implies that CT082, albeit being a T3S substrate, may not be an effector. First, the 3D structure of Cpn0803 (the ortholog of CT584 in *C. pneumoniae*) is unique [51]. Second, Cpn0803 was shown to form hexamers in solution [51], which has not been described for T3S chaperones. Third, CT584 does not bind to the N-terminal region of its substrate but instead to a central part of CT082 (Fig. 5). Differently from class I chaperones, class II and class III chaperones can have features different from other T3S chaperones [68]–[73]. For example, CesA, the T3S chaperone of the *E. coli* EspA filament protein, binds to two discrete regions within the N- and C-terminus of EspA and its 3D structure does not resemble that of other classic T3S chaperones [71]. Because class II chaperones are dedicated to pore-forming translocators, and the corresponding putative chlamydial translocators have been identified [14], CT584 could be a class III chaperone. If that is the case, CT082 may be involved in the assembly of the injectisome or part of a related substructure. However, at the present, we cannot fully discard that CT584 could be a chaperone of effectors as T3SSs may have evolved differently in *Chlamydiae* than in Proteobacteria. We are currently analyzing these possibilities by examining the subcellular localization of CT082 in *C. trachomatis* infected cells and testing if it can interact with components of the *Chlamydia* T3SS.

A characteristic of CT082 is a long region (between amino acids 319 and 456) predicted to be intrinsically unstructured, as analyzed with PredictProtein (www.predictprotein.org) [74]. These no regular secondary structure (NORS) regions are widespread in nature and this apparent lack of structure is crucial for protein function [75]. These regions are often involved in protein-protein interactions [75], and the one in CT082 is within the identified binding region of CT584 (amino acids 330–450). Therefore, it is likely that CT584 protects a region of CT082 that makes it unstable (Fig. 6) in the absence of its target(s) but which should be important for its function.

In summary, we have identified novel elements of the *C. trachomatis* T3S system. We also revealed CT584 as a T3S chaperone with unique characteristics. Our work suggests hypotheses for a role of Slc1 as a regulator of early effector secretion and of CT082 as being involved in the assembly of the *C. trachomatis* injectisome.

## Supporting Information

Figure S1 **Slc1 interacts with Tarp, CT694, and CT695, and CT584 interacts with CT082.** Overall immunoblot images corresponding to the results presented in Fig. 2A (A) and in Fig. 4A (B). *Y. enterocolitica* ΔHOPEMT strains expressing the indicated proteins were grown in non-secreting conditions. The bacterial cells were lysed and proteins in the lysate supernatants (input) were immunoprecipitated with mouse monoclonal anti-Myc antibodies bound to Protein G agarose beads (output). The input (Inp.) and output (Outp.) fractions from the immunoprecipitations (IPs) were analyzed by immunoblotting with rabbit polyclonal anti-Myc and rat monoclonal anti-HA antibodies.
(PDF)

Figure S2 **Slc1 interacts with Tarp, CT694 and CT695 (A), and CT584 interacts with CT082 (B).** *Y. enterocolitica* ΔHOPEMT strains expressing the indicated proteins were grown in T3S-inducing conditions (without Ca^2+^). The bacterial cells were lysed and proteins in the lysate supernatants (input) were immunoprecipitated with mouse monoclonal anti-HA antibodies bound to Protein G agarose beads (output). The input (Inp.) and output (Outp.) fractions from the immunoprecipitations (IPs) were analyzed by immunoblotting with rabbit polyclonal anti-Myc and rat monoclonal anti-HA antibodies.
(PDF)

Figure S3 **Expression of CT082 and CT584 during host cell infection by** ***C. trachomatis*** **.** (A and B) To analyze the specificity of rabbit polyclonal anti-CT082 (α-CT082) (A) and anti-CT584 (α-CT584) (B) antibodies, HeLa HtTA1 cells were transfected with plasmids encoding EGFP, EGFP-CT082, CT082-HA, and CT584-HA (as indicated). Extracts from the transfected cells were analyzed by immunoblotting, comparing α-CT082 and α-CT584 antibodies relative to commercial α-GFP and α-HA antibodies, respectively. (C and D) HeLa 229 cells were left uninfected (UI) or infected with C. trachomatis L2/434 for the indicated times. Protein extracts were analyzed by immunoblotting with α-CT082, α-CT584, α-*C. trachomatis* major outer membrane protein (MOMP), and α-tubulin (loading control) antibodies.
(PDF)

Table S1 **Plasmids used and constructed in this study.**
(PDF)

Table S2 **Primers used in this work.**
(PDF)
