# Supplementary material for: Identification of Novel Type III Secretion Chaperone-Substrate Complexes of Chlamydia trachomatis
Source: PLoS One. 2013 Feb 19;8(2):e56292. doi: 10.1371/journal.pone.0056292 (PMC3576375; doi:10.1371/journal.pone.0056292)
Supplement: Figure S1 — Slc1 interacts with Tarp, CT694, and CT695, and CT584 interacts with CT082. Overall immunoblot images corresponding to the results presented in Fig. 2A (A) and in Fig. 4A (B). Y. enterocolitica ΔHOPEMT strains expressing the indicated proteins were grown in non-secreting conditions. The bacterial cells were lysed and proteins in the lysate supernatants (input) were immunoprecipitated with mouse monoclonal anti-Myc antibodies bound to Protein G agarose beads (output). The input (Inp.) and output (Outp.) fractions from the immunoprecipitations (IPs) were analyzed by immunoblotting with rabbit polyclonal anti-Myc and rat monoclonal anti-HA antibodies. (PDF) [file pone.0056292.s001.pdf]

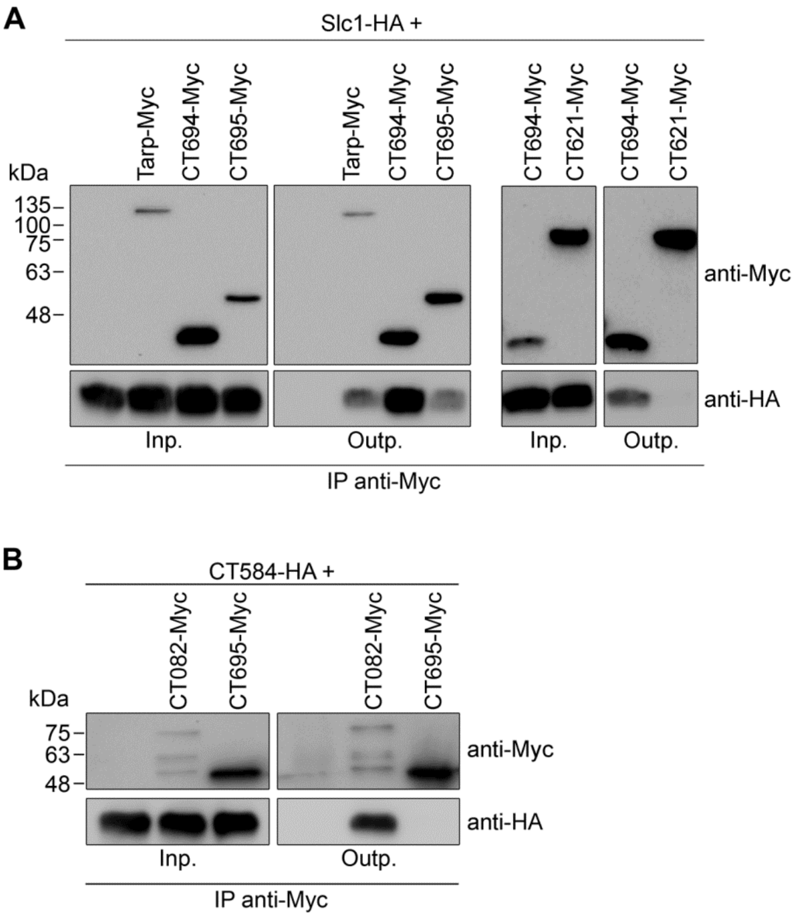

Figure S1. Slc1 interacts with Tarp, CT694, and CT695, and CT584 interacts with CT082. Overall immunoblot images corresponding to the results presented in Fig. 2A (A) and in Fig 4A (B). *Y. enterocolitica* ΔHOPEMT strains expressing the indicated proteins were grown in non-secreting conditions. The bacterial cells were lysed and proteins in the lysate supernatants (input) were immunoprecipitated with mouse monoclonal anti-Myc antibodies bound to Protein G agarose beads (output). The input (Inp.) and output (Outp.) fractions from the immunoprecipitations (IPs) were analyzed by immunoblotting with rabbit polyclonal anti-Myc and rat monoclonal anti-HA antibodies.
