# Supplementary material for: Identification of Novel Type III Secretion Chaperone-Substrate Complexes of Chlamydia trachomatis
Source: PLoS One. 2013 Feb 19;8(2):e56292. doi: 10.1371/journal.pone.0056292 (PMC3576375; doi:10.1371/journal.pone.0056292)
Supplement: Figure S2 — Slc1 interacts with Tarp, CT694 and CT695 (A), and CT584 interacts with CT082 (B). Y. enterocolitica ΔHOPEMT strains expressing the indicated proteins were grown in T3S-inducing conditions (without Ca2+). The bacterial cells were lysed and proteins in the lysate supernatants (input) were immunoprecipitated with mouse monoclonal anti-HA antibodies bound to Protein G agarose beads (output). The input (Inp.) and output (Outp.) fractions from the immunoprecipitations (IPs) were analyzed by immunoblotting with rabbit polyclonal anti-Myc and rat monoclonal anti-HA antibodies. (PDF) [file pone.0056292.s002.pdf]

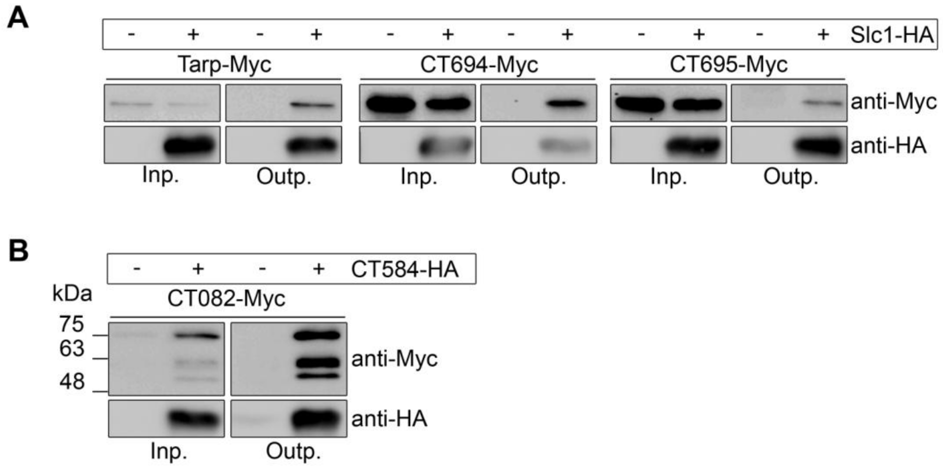

Figure S2. Slc1 interacts with Tarp, CT694 and CT695 (A), and CT584 interacts with CT082 (B). *Y. enterocolitica* ΔHOPEMT strains expressing the indicated proteins were grown in T3S-inducing conditions (without Ca^{2+}). The bacterial cells were lysed and proteins in the lysate supernatants (input) were immunoprecipitated with mouse monoclonal anti-HA antibodies bound to Protein G agarose beads (output). The input (Inp.) and output (Outp.) fractions from the immunoprecipitations (IPs) were analyzed by immunoblotting with rabbit polyclonal anti-Myc and rat monoclonal anti-HA antibodies.
