# Supplementary material for: Identification of Novel Type III Secretion Chaperone-Substrate Complexes of Chlamydia trachomatis
Source: PLoS One. 2013 Feb 19;8(2):e56292. doi: 10.1371/journal.pone.0056292 (PMC3576375; doi:10.1371/journal.pone.0056292)
Supplement: Figure S3 — Expression of CT082 and CT584 during host cell infection by C. trachomatis . (A and B) To analyze the specificity of rabbit polyclonal anti-CT082 (α-CT082) (A) and anti-CT584 (α-CT584) (B) antibodies, HeLa HtTA1 cells were transfected with plasmids encoding EGFP, EGFP-CT082, CT082-HA, and CT584-HA (as indicated). Extracts from the transfected cells were analyzed by immunoblotting, comparing α-CT082 and α-CT584 antibodies relative to commercial α-GFP and α-HA antibodies, respectively. (C and D) HeLa 229 cells were left uninfected (UI) or infected with C. trachomatis L2/434 for the indicated times. Protein extracts were analyzed by immunoblotting with α-CT082, α-CT584, α-C. trachomatis major outer membrane protein (MOMP), and α-tubulin (loading control) antibodies. (PDF) [file pone.0056292.s003.pdf]

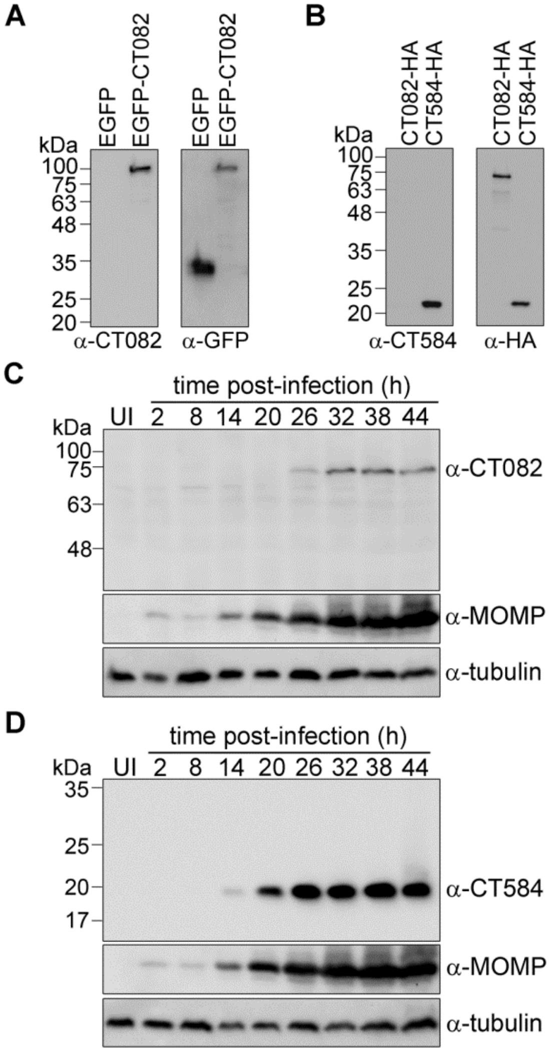

Figure S3. Expression of CT082 and CT584 during host cell infection by *C. trachomatis*. (A and B) To analyze the specificity of rabbit polyclonal anti-CT082 (α-CT082) (A) and anti-CT584 (α-CT584) (B) antibodies, HeLa HtTA1 cells were transfected with plasmids encoding EGFP, EGFP-CT082, CT082-HA, and CT584-HA (as indicated). Extracts from the transfected cells were analyzed by immunoblotting, comparing α-CT082 and α-CT584 antibodies relative to commercial α-GFP and α-HA antibodies, respectively. (C and D) HeLa 229 cells were left uninfected (UI) or infected with C. trachomatis L2/434 for the indicated times. Protein extracts were analyzed by immunoblotting with α-CT082, α-CT584, α-*C. trachomatis* major outer membrane protein (MOMP), and α-tubulin (loading control) antibodies.
