# Supplementary material for: Identification of Novel Type III Secretion Chaperone-Substrate Complexes of Chlamydia trachomatis
Source: PLoS One. 2013 Feb 19;8(2):e56292. doi: 10.1371/journal.pone.0056292 (PMC3576375; doi:10.1371/journal.pone.0056292)
Supplement: Table S1 — Plasmids used and constructed in this study. (PDF) [file pone.0056292.s004.pdf]

Table S1. Plasmids used and constructed in this study.

Plasmid	Description	Reference or source
pUT18	BACTH vector for fusions to the N-terminus of fragment T18	Euromedex
pUT18C	BACTH vector for fusions to the C-terminus of fragment T18	Euromedex
pKNT25	BACTH vector for fusions to the N-terminus of fragment T25	Euromedex
pKT25	BACTH vector for fusions to the C-terminus of fragment T25	Euromedex
pKT25-zip	Positive control for BACTH assays; encodes Zip-T25	Euromedex
pUT18C-zip	Positive control for BACTH assays; encodes Zip-T18	Euromedex
pBAD/*Myc*-His A	Cloning and expression vector	Invitrogen
pBBR1-MCS2	Low-copy cloning vector	[1]
pLJM3	Low copy vector, derivative of pBBR1MCS-234. Expresses YopE under the control of its own promoter (P*yopE*).	[2]
pCDFDuet-1	Expression vector for the co-expression of two targets ORFs	Novagen
pGEX-4T-2	Expression vector to generate fusions to the C-terminus of glutathione S-transferase (GST)	GE Healthcare
pMal-c	Expression vector to generate fusions to the C-terminus of maltose binding protein (MBP)	New England Biolabs
pEGFP-c1	Transfection vector	Clontech
pcDNA6/V5-His A	Transfection vector	Invitrogen
pEF6/*Myc*-His A	Transfection vector	Invitrogen
pSVP15	*ct043*/*slc1* cloned into pKNT25; encodes Slc1-T25	This study
pSVP16	*ct043*/*slc1* cloned into pUT18; encodes Slc1-T18	This study
pSVP17	*ct260*/*mscs* cloned into pKNT25; encodes Mscs-T25	This study
pSVP34	*ct260*/*mscs* cloned into pUT18; encodes Mscs-T18	This study
pSVP54	*ct274* cloned into pKT25; encodes T25-CT274	This study
pSVP55	*ct274* cloned into pUT18C; encodes T18-CT274	This study
pSVP24	*ct584* cloned into pKNT25; encodes CT584-T25	This study
pSVP41	*ct584* cloned into pUT18; encodes CT584-T18	This study
pSVP56	*ct663*/*scc4* cloned into pKT25; encodes T25-Scc4	This study
pSVP57	*ct663*/*scc4* cloned into pUT18C; encodes T18-Scc4	This study
pSVP31	*ct790* cloned into pKNT25; encodes CT790-T25	This study
pSVP46	*ct790* cloned into pUT18; encodes CT790-T18	This study
pSVP33	*ct845* cloned into pKNT25; encodes CT845-T25	This study
pSVP48	*ct845* cloned into pUT18; encodes CT845-T18	This study
pSVP66	*ct456*/*tarp* cloned into pUT18C; encodes T18-Tarp	This study
pSVP72	*ct694* cloned into pUT18C; encodes T18-CT694	This study
pSVP73	*ct695* cloned into pUT18C; encodes T18-CT695	This study
pSVP119	*ct082* cloned into pUT18C; encodes T18-CT082	This study
pSVP115	*ct619* cloned into pKNT25; encodes CT619-T25	This study
pSVP117	*ct620* cloned into pKNT25; encodes CT620-T25	This study
pSVP131	*ct621* cloned into pKNT25; encodes CT621-T25	This study
pSVP136	*ct711* cloned into pKNT25; encodes CT711-T25	This study
pSVP137	*ct712* cloned into pKNT25; encodes CT712-T25	This study
pSVP75	*ct621* cloned into pUT18C; encodes T18-CT621	This study
pSVP138	*ct043*/*slc1-HA* cloned into pBBR1-MCS2 under the control of the *Y. enterocolitica sycE* promoter; encodes Slc1-HA	This study
pSVP85	*ct456*/*tarp* cloned into pBAD/*Myc*-His A; encodes Tarp-Myc	This study
pSVP87	*ct694* cloned into pBAD/*Myc*-His A; encodes CT694-Myc	This study
pSVP88	*ct695* cloned into pBAD/*Myc*-His A; encodes CT695-Myc	This study
pSVP141	*ct621* cloned into pBAD/*Myc*-His A; encodes CT621-Myc	This study
pSVP186	$tarp_{1\text{-}100}$ cloned into pBAD/*Myc*-His A; encodes $\text{Tarp}_{1\text{-}100}$-Myc	This study

Table S1. *Continued.*

Plasmid	Description	Reference or source
pSVP187	*tarp*$_{101\text{-}1001}$ cloned into pBAD/*Myc*-His A; encodes Tarp$_{101\text{-}1001}$-Myc	This study
pSVP190	*ct694*$_{1\text{-}132}$ cloned into pBAD/*Myc*-His A; encodes CT694$_{1\text{-}132}$-Myc	This study
pSVP197	*ct694*$_{133\text{-}322}$ cloned into pBAD/*Myc*-His A; encodes CT694$_{133\text{-}322}$-Myc	This study
pSVP193	*ct695*$_{1\text{-}103}$ cloned into pBAD/*Myc*-His A; encodes CT695$_{1\text{-}104}$-Myc	This study
pSVP194	*ct694*$_{104\text{-}398}$ cloned into pBAD/*Myc*-His A; encodes CT695$_{104\text{-}398}$-Myc	This study
pSVP145	*ct584-HA* cloned into pBBR1-MCS2 under the control of the *Y. enterocolitica sycE* promoter; encodes CT584-HA	This study
pSVP142	*ct082* cloned into pBAD/*Myc*-His A; encodes CT082-Myc	This study
pCM24	*ct082* cloned into pGEX-4T-2; encodes GST-CT082	This study
pSVP182	gst-ct0821-100 cloned into pCDFDuet-1; encodes GST-CT0821-100	This study
pSVP183	*gst-ct082*$_{101\text{-}560}$ cloned into pCDFDuet-1; encodes GST-CT082$_{101\text{-}560}$	This study
pSVP184	*gst-ct082*$_{1\text{-}200}$ cloned into pCDFDuet-1; encodes GST-CT082$_{1\text{-}200}$	This study
pSVP185	*gst-ct082*$_{201\text{-}560}$ cloned into pCDFDuet-1; encodes GST-CT082$_{201\text{-}560}$	This study
pSVP200	*ct082*$_{281\text{-}560}$ cloned into pGEX-4T-2; encodes GST-CT082$_{281\text{-}560}$	This study
pSVP201	*ct082*$_{331\text{-}560}$ cloned into pGEX-4T-2; encodes GST-CT082$_{331\text{-}560}$	This study
pSVP202	*ct082*$_{451\text{-}560}$ cloned into pGEX-4T-2; encodes GST-CT082$_{451\text{-}560}$	This study
pSVP203	*ct082*$_{331\text{-}450}$ cloned into pGEX-4T-2; encodes GST-CT082$_{331\text{-}450}$	This study
pSVP206	*ct082*$_{\Delta 330\text{-}450}$ cloned into pGEX-4T-2; encodes GST-CT082$_{\Delta 330\text{-}450}$	This study
pSVP90	*yopE* cloned into pBAD/*Myc*-His A; encodes YopE-Myc	This study
pFA17	*ct043/slc1-HA* cloned into pLJM3; encodes Slc1-HA	This study
pSVP82	*ct584HA* cloned into pLJM3; encodes CT584-HA	This study
pSVP83	*ct790-HA* cloned into pLJM3; encodes CT790-HA	This study
pSVP147	*ct584* cloned into pCDFDuet-1; encodes (6x)His-CT584	This study
pCM25	*ct082* cloned into pMal-c; encodes MBP -CT082	This study
pFA105	*ct082-HA* cloned into pcDNA/V5-His A; encodes CT082-HA	This study
pSG13	*ct082* cloned into pEGFP-c1; encodes EGFP-CT082	This study
pSVP198	*ct584-HA* cloned into pEF6/*Myc*-His A; encodes CT584-HA	This study

[a][1] Kovach ME, Elzer PE, Hill DS, Robertson GT, Farris MA, et al. (1995) Four new derivatives of the broad-host-range cloning vector pBBR1MCS, carrying different antibiotic-resistance cassettes. Gene 166: 175-176; [2] Marenne MN, Journet L, Mota LJ, Cornelis GR (2003) Genetic analysis of the formation of the Ysc-Yop translocation pore in macrophages by *Yersinia enterocolitica*: role of LcrV, YscF and YopN. Microb Pathog 35: 243-258.
