# Supplementary material for: Identification of Novel Type III Secretion Chaperone-Substrate Complexes of Chlamydia trachomatis
Source: PLoS One. 2013 Feb 19;8(2):e56292. doi: 10.1371/journal.pone.0056292 (PMC3576375; doi:10.1371/journal.pone.0056292)
Supplement: Table S2 — Primers used in this work. (PDF) [file pone.0056292.s005.pdf]

Table S2. Primers used in this work.

Code	Description	[a]Sequence (5'-3')	Restriction enzyme
279	Forward primer to construct pSVP15 and pSVP16	GATCCTGCAGGTCCAGGCAGAATGCTGAGG	*Pst*I
280	Reverse primer to construct pSVP15 and pSVP16	GATCGGATCCTCTGCACGGATTCCTGCTGGAG	*Bam*HI
285	Forward primer to construct pSVP17 and pSVP34	GATCAAGCTTGACAACGTGGACTTTGAATCAC	*Hind*III
286	Reverse primer to construct pSVP17 and pSVP34	GATCGGATCCTCAGGCTCTAGCTGATCGGATTG	*Bam*HI
331	Foward primer to construct pSVP54 and pSVP55	GATCGGATCCCTTAGATAATGAGTGGAAAGC	*Bam*HI
332	Reverse primer to construct pSVP54 and pSVP55	GATCGGTACCCGCATAACTAAGTAGTAATGC	*Kpn*I
299	Forward primer to construct pSVP24 and pSVP41	GATCAAGCTTGACGACGAAACCCAAAACTCTAG	*Hind*III
300	Reverse primer to construct pSVP24 and pSVP41	GATCGGATCCTCCACAGATTTCGTTAATTCTTC	*Bam*HI
333	Foward primer to construct pSVP56	GATCCTGCAGGGTTGGAAAAATTGATAAAGAATTTTG	*Pst*I
334	Foward primer to construct pSVP57	GATCCTGCAGGTTGGAAAAATTGATAAAGAATTTTG	*Pst*I
335	Reverse primer to construct pSVP56 and pSVP57	GATCGGTACCCGCTCCTGCTCCGTTTTACTCAG	*Kpn*I
315	Forward primer to construct pSVP31 and pSVP46	GATCAAGCTTGGATAAGCAAAGATTAAAATTAG	*Hind*III
316	Reverse primer to construct pSVP31 and pSVP46	GATCGGATCCTCTTCTTCAGTTTGAATCACAG	*Bam*HI
319	Forward primer to construct pSVP33 and pSVP48	GATCAAGCTTGAAGGAAGAAATTCTCGCGC	*Hind*III
320	Reverse primer to construct pSVP33 and pSVP48	GATCGGATCCTCATCCTTACAGAGGCTCGTATCG	*Bam*HI
354	Forward primer to construct pSVP66	GATCGGATCCCACGAATTCTATATCAGGTGATC	*Bam*HI
355	Reverse primer to construct pSVP66	GATCGGTACCCGTCCTACGGTATCAATCAGTGAGC	*Kpn*I
367	Forward primer to construct pSVP72	GATCGGATCCCAGTATTCGACCTACTAATGG	*Bam*HI
368	Reverse primer to construct pSVP72	GATCGGTACCCGGTCTAAGAAAACAGAAGAAG	*Kpn*I
369	Forward primer to construct pSVP73	GATCGGTACCGAGTAGCATAAGCCCTATAGG	*Kpn*I
370	Reverse primer to construct pSVP73	GATCGGTACCCGGATATTCCCAACCGAAGAAGG	*Kpn*I
563	Forward primer to construct pSVP119	GATCGGTACCGTCAATTTCTGGAAGTGGTAATGTATC	*Kpn*I
564	Reverse primer to construct pSVP119	GATCGAATTCGATGAATCGCCGCCTGCATCC	*Eco*RI
359	Forward primer to construct pSVP115	GATCGGATCCCTCTTCTTACTATTTAAATTTTAGGC	*Bam*HI
360	Reverse primer to construct pSVP115	GATCGAATTCGAATTAGAATAAATTTCCGCTGC	*Eco*RI

Table S2. ***Continued.***

Code	Description	[a]Sequence (5’-3’)	Restriction enzyme
361	Forward primer to construct pSVP117	GATCGGATCCCTGTTCTATGAACATATTTAATAAAATTAAC	*Bam*HI
362	Reverse primer to construct pSVP117	GATCGGTACCCGACTAGCCAGTTTTCTTGTTAAACC	*Kpn*I
363	Forward primer to construct pSVP131 and pSVP75	GATCGGATCCCAACCGTATTCATCGTACACAAGG	*Bam*HI
364	Reverse primer to construct pSVP131 and pSVP75	GATCGGTACCCGTCTTAAGAGATTACGCGC	*Kpn*I
371	Forward primer to construct pSVP136	GATCGGATCCCTCAATACAACCTACATCCATTTC	*Bam*HI
372	Reverse primer to construct pSVP136	GATCGGTACCCGTTTAAATCTACGGATCAACTTAGC	*Kpn*I
373	Forward primer to construct pSVP137	GATCGGATCCCAGAAACCATCCGATTCCAGAAGG	*Bam*HI
374	Reverse primer to construct pSVP137	GATCGAATTCGAGCTAGAAGCCAATGTTCTATATAC	*Eco*RI
28	Forward primer to construct pSVP138 and pSVP145	GATTAAGTTGGGTAACGCC	
55	Reverse primer to construct pSVP138 and pFA17	GATCCTCGAGTTAAGCATAATCAGGAACATCATACGGAT	*Xho*I
782	Overlapping forward primer to construct pSVP138	ATGCACGGATTCCTGCTGGAGGGGTTAAGTTGATATTTTATCTAACTATTAGGAGATCAAATGTCCAGGCAGAATGCTGAGG	
783	Overlapping reverse primer to construct pSVP138	CCTCAGCATTCTGCCTGGACATTTGATCTCCTAATAGTTAGATAAAATATCAACTTAACC	
5	Forward primer to construct pSVP85 and pSVP186	GATCTCATGACGAATTCTATATCAGGTGATCAACCTACTG	*Bsp*HI
460	Reverse primer to construct pSVP85 and pSVP187	GATCGGTACCAGTCCTACGGTATCAATCAGTGAGC	*Kpn*I
461	Forward primer to construct pSVP87 and pSVP190	GATCTCATGAGTATTCGACCTACTAATGG	*Bsp*HI
462	Reverse primer to construct pSVP87 and pSVP197	GATCGGTACCAGGTCTAAGAAAACAGAAGAAG	*Kpn*I
463	Forward primer to construct pSVP88 and pSVP193	GATCTCATGAGTAGCATAAGCCCTATAGG	*Bsp*HI
464	Reverse primer to construct pSVP88 and pSVP194	GATCGGTACCAGGATATTCCCAACCGAAGAAGG	*Kpn*I
772	Forward primer to construct pSVP141	GATCTCATGAACCGTATTCATCGTAC	*Bsp*HI
773	Reverse primer to construct pSVP141	GATCGGTACCAGTCTTAAGAGATTACGCGCTAATCC	*Kpn*I
1060	Reverse primer to construct pSVP186	GATCGGTACCAGTCCAGCAACGGCTTGGGAAG	*Kpn*I
1061	Forward primer to construct pSVP187	GATCTCATGAAAATCACTTCTCCAGATAC	*Bsp*HI
1064	Reverse primer to construct pSVP190	GATCGGTACCAGTTGTCCCTCCATAGAACCTAGC	*Kpn*I
1065	Forward primer to construct pSVP197	GATCTCATGAGTCCTCAAGATTATAAAGC	*Bsp*HI

Table S2. *Continued.*

Code	Description	[a]Sequence (5'-3')	Restriction enzyme
1068	Reverse primer to construct pSVP193	GATCGGTACCAGATTCCTTCGTGCAACACGACG	*Kpn*I
1069	Forward primer to construct pSVP194	GATCCCATGGAAAACTATGATGTGAAAAAAGC	*Nco*I
466	Reverse primer to construct pSVP145 and pSVP82	GATCAAGCTTAAGCATAATCAGGAACATCATACGGATACACAGATTTCGTTAATTCTTC	*Hind*III
784	Overlapping forward primer to construct pSVP145	GGTTAAGTTGATATTTTATCTAACTATTAGGAGATCAAATGACGACGAAACCCAAAACTCTAG	
785	Overlapping reverse primer to construct pSVP145	CTAGAGTTTTGGGTTTCGTCGTCATTTGATCTCCTAATAGTTAGATAAAATATCAACTTAACC	
774	Forward primer to construct pSVP142	GATCTCATGAGTATTTCTGGAAGTGGTAATG	*Bsp*HI
775	Reverse primer to construct pSVP142	GATCGGTACCAGTGAATCGCCGCCTGCATCC	*Kpn*I
796	Forward primer to construct pCM24 and pSVP206	GATCCGAATTCCCTCAATTTCTGGAAGTGGTAATG	*Eco*RI
787	Reverse primer to construct pCM24, pSVP200, pSVP201, pSVP202, pSVP206, pCM25	GATCGTCGACTCATGAATCGCCGCCTGCATCC	*Sal*I
742	Forward primer to construct pSVP182, pSVP183, pSVP184 and pSVP185	GGGAATTCCATATGTCCCCTATACTAGGTTATTGG	*Nde*I
1072	Reverse primer to construct pSVP182	GATCGGTACCTCATCCTTTAGTACCTGCACGTAC	*Kpn*I
755	Reverse primer to construct pSVP183 and pSVP185	GATCGGTACCTCATGAATCGCCGCCTGCATCC	*Kpn*I
1073	Overlapping forward primer to construct pSVP183	CGACTCGAGCGGCCGCATCGTTCCTTACAGGATTTGGAACG	
1074	Overlapping reverse primer to construct pSVP183	CGTTCCAAATCCTGTAAGGAACGATGCGGCCGCTCGAGTCG	
1075	Reverse primer to construct pSVP184	GATCGGTACCTCATGCAGTGATTTCTCCAGATG	*Kpn*I
1076	Overlapping forward primer to construct pSVP185	CGACTCGAGCGGCCGCATCGTTAGCTTCGTTTAATCCTAATGTTCG	
1077	Overlapping reverse primer to construct pSVP185	CGAACATTAGGATTAAACGAAGCTAACGATGCGGCCGCTCGAGTCG	
1178	Forward primer to construct pSVP200	GATCGAATTCCCGATCCAGCTTCTGCAGAAG	*Eco*RI
1179	Forward primer to construct pSVP201 and pSVP203	GATCGAATTCCCCAATTTCCTGAACCTAAGTTC	*Eco*RI
1180	Forward primer to construct pSVP202	GATCGAATTCCCCCTTCCCATTTAGGGATGGC	*Eco*RI
1181	Reverse primer to construct pSVP203	GATCCTCGAGTCAAAACTGGTAAGCCGAACTAACCG	*Xho*I
1190	Overlapping forward primer to construct pSVP206	CCACTCCTAGTTCAGGCTCATTTCCTTCCCATTTAGGGATGG	
1191	Overlapping reverse primer to construct pSVP206	CCATCCCTAAATGGGAAGGAAATGAGCCTGAACTAGGAGTGG	

Table S2. ***Continued.***

Code	Description	[a]Sequence (5'-3')	Restriction enzyme
484	Forward primer to construct pFA105	GATCGGTACCATGGCAATTTCTGGAAGTGGTAATG	*EcoR*I
29	Reverse primer to construct pFA105	TTGTGTGGAATTGTGAGCG	
650	Forward primer to construct pSG13	GATCCTCGAGCTTCAATTTCTGGAAGTGG	*Xho*I
651	Reverse primer to construct pSG13	GATCGGATCCTCATGAATCGCCGCCTGCATCC	*Bam*HI
1098	Forward primer to construct pSVP198	GATCGGATCCACAATGGACACGAAACCCAAAACTCTAGA	*Bam*HI
1099	Reverse primer to construct pSVP198	ATAAGAATGCGGCCGCTTAAGCATAATCAGGAACATCAT	*Not*I
435	Forward primer to construct pSVP90	GATCTCATGAAAATATCATCATTTATTTCTACATCACTGC	*Bsp*HI
436	Reverse primer to construct pSVP90	GATCGGTACCAGCATCAATGACAGTAATTGC	*Kpn*I
54	Forward primer to construct pFA17	GATTTCCATATGTCCAGGCAGAATGCTG	*Nde*I
465	Forward primer to construct pSVP82	GGGAATTCCATATGACGACGAAACCCAAAACTCTAG	*Nde*I
467	Forward primer to construct pSVP83	GGGAATTCCATATGGATAAGCAAAGATTAAAATTAG	*Nde*I
468	Reverse primer to construct pSVP83	GATCAAGCTTAAGCATAATCAGGAACATCATACGGATATTCTTCAGTTTGAATCACAG	*Hind*III
738	Forward primer to construct pSVP147	GATCGGATCCGACGACGAAACCCAAAACTC	*Bam*HI
739	Reverse primer to construct pSVP147	GATCAAGCTTCACAGATTTCGTTAATTCTTC	*Hind*III
786	Forward primer to construct pCM25	GATCCGAATTCTCAATTTCTGGAAGTGGTAATG	*Eco*RI

[a]Sites of restriction enzymes are underlined
